# Pronounced Plastic and Evolutionary Responses to Unpredictable Thermal Fluctuations in *Drosophila simulans*

**DOI:** 10.3389/fgene.2020.555843

**Published:** 2020-10-28

**Authors:** Jesper G. Sørensen, Tommaso Manenti, Jesper S. Bechsgaard, Mads F. Schou, Torsten N. Kristensen, Volker Loeschcke

**Affiliations:** ^1^Department of Biology, Aarhus University, Aarhus, Denmark; ^2^Department of Biology, Lund University, Lund, Sweden; ^3^Department of Chemistry and Bioscience, Aalborg University, Aalborg, Denmark

**Keywords:** heat tolerance, genomics, proteomics, thermal fluctuations, *Drosophila simulans*

## Abstract

Organisms are exposed to temperatures that vary, for example on diurnal and seasonal time scales. Thus, the ability to behaviorally and/or physiologically respond to variation in temperatures is a fundamental requirement for long-term persistence. Studies on thermal biology in ectotherms are typically performed under constant laboratory conditions, which differ markedly from the variation in temperature across time and space in nature. Here, we investigate evolutionary adaptation and environmentally induced plastic responses of *Drosophila simulans* to no fluctuations (constant), predictable fluctuations or unpredictable fluctuations in temperature. We whole-genome sequenced populations exposed to 20 generations of experimental evolution under the three thermal regimes and examined the proteome after short-term exposure to the same three regimes. We find that unpredictable fluctuations cause the strongest response at both genome and proteome levels. The loci showing evolutionary responses were generally unique to each thermal regime, but a minor overlap suggests either common laboratory adaptation or that some loci were involved in the adaptation to multiple thermal regimes. The evolutionary response, i.e., loci under selection, did not coincide with induced responses of the proteome. Thus, genes under selection in fluctuating thermal environments are distinct from genes important for the adaptive plastic response observed within a generation. This information is key to obtain a better understanding and prediction of the effects of future increases in both mean and variability of temperatures.

## Introduction

It is well known that different adaptive responses for coping with stressful temperature conditions exist. Within generations, organisms can respond plastically to environmental changes (Pigliucci, 1996, 2001; Lande, 2009), while evolutionary responses may occur through both changes in trait means and in the level of plasticity (Hoffmann and Parsons, 1989; Williams et al., 2008; Lande, 2009; Kristensen et al., 2018). It is debated whether plasticity or evolutionary responses constitute the main contributor to temperature adaptation in small ectothermic animals (Gunderson and Stillman, 2015; Sgrò et al., 2016; Sørensen et al., 2016a). Evolutionary change in trait means is better understood and does provide evidence for local adaptation (e.g., Hoffmann et al., 2002; Kellermann et al., 2012). However, upper thermal limits seem to be evolutionary constrained in some small ectothermic insects (particularly studied in *Drosophila*) (Kellermann et al., 2012; Schou et al., 2014; but see discussion in Logan and Cox, 2020), while not in some species of phytoplankton (Kontopoulos et al., 2020). The constraint among species of *Drosophila* is not founded in an apparent lack of additive genetic variation, as significant levels of genetic variation for heat tolerance in the same species of *Drosophila* have been documented (Williams et al., 2012; Castaneda et al., 2019). Theory predicts that evolution of plasticity should be favored in predictably variable environments (Lande, 2009; Ashander et al., 2016). However, for plasticity in upper (and lower) thermal tolerance empirical evidence supporting this hypothesis is scarce (Gunderson and Stillman, 2015). Thus, it is not clear how and how much small ectothermic animals can and will respond to a warming climate.

Under natural conditions, organisms are exposed to temperatures that vary on diurnal (multiple exposures within a generation for most insect species) and seasonal (often across multiple generations in insects) scales. Diurnal fluctuations are often comparable to seasonal variation in magnitude, but characterized by temperature changes which occur much faster than temperature change across seasons. For example, according to the Danish Meteorological Institute, the difference in mean temperature among seasons in Denmark is ∼15°C (based on monthly mean temperatures 2006–2015^1^). Diurnal temperatures can attain a similar range between a cold night and a warm summer day, where 10 and 25°C, respectively, can be found within the same or a few days. The variability in temperature is expected to further increase with increasing heat waves under climate change (Perkins-Kirkpatrick and Lewis, 2020). Under these conditions, it can be questioned whether evolutionary change in trait means is adequate to maintain fitness sufficient high for a species to persist (Araujo et al., 2013; Radchuk et al., 2019). Phenotypic plasticity has been suggested to be more likely to accommodate rapidly changing temperatures and extreme events (Gienapp et al., 2008; Merilä and Hendry, 2014). However, while large differences among *Drosophila* species in plasticity of different traits exist, within species plasticity in heat tolerance (thermal acclimation capacity) is evolving slowly; plasticity does not to differ among geographically distinct populations of the same species, and is not lost when natural populations are kept at constant temperature in the laboratory (up to 28 generations) (Overgaard et al., 2011; van Heerwaarden et al., 2014; Fragata et al., 2016; Manenti et al., 2017). Furthermore, plasticity does not change under experimental evolution (for 20 generations) in thermal regimes with different variability and predictability (Manenti et al., 2015). An explanation for this could be constraints bounded in the genetic architecture of basal and acclimation trait values (Gerken et al., 2015; see also Lecheta et al., 2020). Temperature fluctuations seem to induce heat tolerance through mechanisms different from mechanisms induced by constant temperatures. For example, thermal fluctuations induce a transcriptomic response that is different from the response induced by differences in mean temperature and from the classic heat stress response induced by increasing thermal stress (Sørensen et al., 2016b; Manenti et al., 2018). However, we have little knowledge on the molecular responses to fluctuating environments, and this limits our understanding of acclimation capacity, evolutionary constraints and trade-offs as well as the costs of induced plastic responses.

A number of evolutionary and ecological studies have recently focused on fitness consequences of short- or long-term variation of temperatures (Ketola et al., 2004; Kingsolver et al., 2009; Hallsson and Bjorklund, 2012; Manenti et al., 2014, 2015; Kellermann et al., 2015; Simões et al., 2020). The use of temperature fluctuations in laboratory experiments has been argued to have a greater ecological relevance compared to constant ones, as they are a better proxy of a natural environment (Boyce et al., 2006; Schreiber, 2010). This study aimed to investigate the molecular responses to environments that differ in amplitude and predictability of daily temperature within the life span of the organism studied. We did this using a well-known insect model system (*Drosophila simulans*), which can easily be collected and manipulated (e.g., applying laboratory natural selection in replicated lines) in the laboratory. To investigate plastic responses to thermal fluctuations we compared the induced proteomic expression profiles among thermal regimes prior to experimental evolution. To investigate evolutionary responses to the thermal regimes we applied full genome sequencing. Specifically, we aimed to identify candidate mechanisms for plastic responses to temperature fluctuations in the proteome, and the genomic patterns of selection responses, of replicate lines exposed to twenty generations of selection in constant, predictable or unpredictable fluctuating thermal regimes. We expect that evolutionary responses act on cis-regulatory elements and will be detected in regions of the genome that encodes proteins inducible by temperature fluctuations (plasticity genes), if inducible and evolved mechanisms of heat tolerance are shared (as predicted if plasticity evolves by genetic assimilation, Pigliucci et al., 2006). Alternatively, evolutionary responses may occur in trans-regulatory elements and observed at genome level will be independent of the proteins induced by temperature fluctuations. Furthermore, we expect that evolutionary responses will be most pronounced in the predictably fluctuating environment if amplitude drives evolution. Alternatively, evolutionary responses will be most pronounced in the unpredictably fluctuating if predictability drives evolution.

## Materials and Methods

### Experimental Animals

**FIGURE 1:**
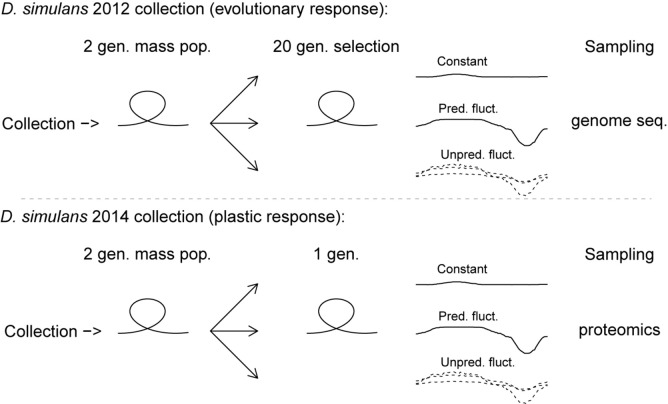
Infographic outlining the design of the study of laboratory responses of *D. simulans* to constant, predictably and unpredictable fluctuating thermal environments. Top part of the figure shows the design of the selection experiment used to evaluate evolutionary responses by genome sequencing. The lower part shows the design of the phenotypic plasticity experiment used to evaluate inducible responses by proteomics. In both experiments the thermal regimes contained independent biological controls. The plotted temperature profiles of the constant, the predictable fluctuating and the unpredictable fluctuating thermal regimes represent the realized cabinet temperatures (temperature data and the thermal regimes are described in more detail in Figure 2).

**FIGURE 2:**
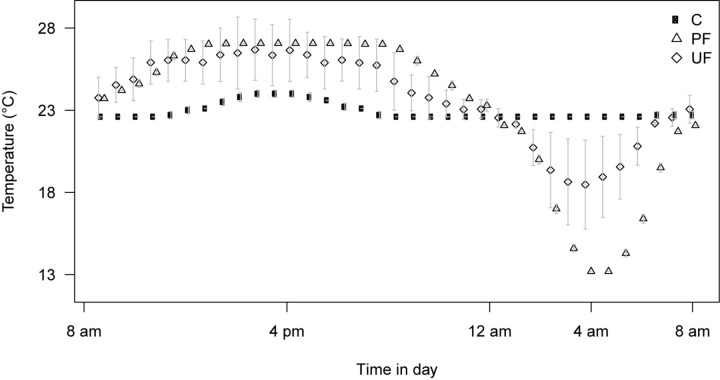
Temperature profile of the three thermal regimes: Constant temperature (C), Predictable fluctuating (PF), and Unpredictable fluctuating (UF). The profiles represent average ± SD for 5 days of recording (points are jittered for better evaluation of error bars). Data and figure modified from Manenti et al. (2014). The average temperature of all regimes is 23°C and flies are exposed to 16:8 h light:dark cycles (note that the light generate a small increase in temperature in the constant regime). The predictable fluctuating regime was programmed to reach 27°C during the light period and 13°C during the dark period, respectively. Low error bars indicate that this was achieved. The unpredictable fluctuating regime was programmed to reach a randomly determined setpoint between 23 and 27°C during the light period and between 23 and 13°C during the dark period, respectively. The average temperatures closer to 23°C and higher SD, respectively, indicate that the fluctuations were on average smaller in amplitude, but unpredictable among days.

Two populations of *D. simulans* both collected at the same field site close to Bologna, Italy, were used in this study. The first was collected in August 2012 (referred to as the collection of ‘*D. simulans* 2012’) and the second was collected at the same field site in August 2014 (referred to as the collection of ‘*D. simulans* 2014’). Flies were throughout maintained in plastic bottles containing 50 mL of standard oatmeal-sugar-yeast-agar *Drosophila* medium at 23°C and a 16:8 h light:dark cycle. All experimental flies were generated using density control by transferring 40–45 eggs to plastic shell-vials with 7 mL medium. The first population (*D. simulans* 2012) was used to investigate the evolutionary response to selection in different thermal regimes, while the second population (*D. simulans* 2014) was used to investigate the plasticity induced by the same regimes, respectively (see Figure 1). The three regimes all had a mean temperature of 23°C, and were either Constant (C), Predictably fluctuating (PF), or Unpredictably fluctuating (UF) in temperature. Custom build programmable thermal cabinets maintained a 16:8 h light:dark cycle, with the C regime maintained at 23°C throughout, while the PF regime followed a 23–28–23°C sine curve during the light phase and a 23–13–23°C sine curve during the dark phase. The UF regime followed the same sine functions, but with a randomly sampled high temperature point between 23 and 28°C during the light phase and a randomly sampled low temperature point between 23 and 13°C during the dark phase (see Figure 2). The thermal regimes are described further in Manenti et al. (2015).

The *D. simulans* 2012 population was used to establish a mass population based on around 350 field caught inseminated females, where after flies were randomly divided into three selection regimes (C, PF, and UF). Each selection regime had three independent biological replicates, each based on three bottles (mixed within replicates each generation) with a combined population size of >500 flies. The selection regimes and maintenance procedures are described further in Manenti et al. (2015). After 20 generations of laboratory natural selection, we froze 250 density controlled females from each biological replicate within each rearing regime, resulting in a total number of 9 samples. These pooled samples were used for full genome sequencing to investigate genomic differences in the three regimes after laboratory experimental evolution.

The *D. simulans* 2014 collection was used to establish a mass population based on 12 bottles of larvae that were offspring of the field collected flies (>500 adult females). This mass population was maintained for two generations in the laboratory at 23°C, before vials with 40 ± 3 eggs were collected and distributed among the three thermal regimes where they developed. Upon emergence, the flies were transferred to fresh food vials and allowed to mature in their respective thermal regimes. When flies were 3–5 days old, flies from 2–3 vials were combined and flash frozen in liquid nitrogen (without anesthesia) to be assayed for the induced response of the proteome. Sampling was performed at 9 am, when the temperature of all three regimes was around 23°C. We sorted females from these samples using a stereomicroscope placed in a 5°C room, with the flies lying on a thin sheet of plastic on top of dry ice to keep the flies as cold as possible. We collected 3 samples of 50 females from each regime (C, PF, and UF, respectively) of *D. simulans* 2014, which were used to investigate the inducible proteomic response to the fluctuating regimes. Furthermore, as the original samples of the founder population before selection collected from the 2012 population were lost (in the initial batch sent for sequencing), we included unselected, density controlled flies collected from *D. simulans* 2014 as an alternative selection control in the full genome sequencing study. For this purpose three replicates of 150 individual females were collected as described above.

### Genome Sequencing, Mapping, and SNP Calling

We extracted DNA from 150 females pooled from each of the 12 lines (three replicates of the founder *D. simulans* 2014 collection, and one sample of each of the three replicated selection lines (C, PF, and UF regimes, respectively) from the *D. simulans* 2012 collection). DNA was Illumina sequenced (100 bp PE) by BGI Hong Kong Co., Limited. The raw reads were quality filtered using TrimGalore^2^ (parameters; –quality 20, –length 75). The filtered reads were mapped to the *D. simulans* reference genome (ASM75419v3^3^) using bwa (version 0.7.5a, mem algorithm, default parameters). Samtools (version 1.6.0) (Li et al., 2009) was used to convert sam to bam files, to sort bam files, to remove duplicates, and to make mpileup files (mpileup −6 –q 20 –d 100). In total 93,821,525 sites were analyzed. The following analyses were done using PoPoolation2 (Kofler et al., 2011); (1) indels were identified and removed (identify-indel-region.pl –min-count 10 –indel-window 5, filter-sync-by-gft.pl), (2) resulting mpileup files were converted to synchronized (sync) files (mpileup2sync.pl –min-qual 20), and (3) downsampled to coverage 40 (downsample-synchronized.pl –target-coverage 40 –max-coverage 10000 –method fraction).

### Genomic Change (Divergence From Base Population)

Using the samples from the unselected ‘*D. simulans* 2014’ as a base population, we estimated consistent allele frequency changes that have occurred during the 20 generations of experimental evolution in the three replicate populations from each of three different thermal selection regimes; constant (C), predictable fluctuating (PF) and unpredictable fluctuating (UF) temperatures. We expect genetic drift to have an equally strong impact across the three selection regimes given that population sizes were kept constant across selection regimes and replicates. Therefore consistent differences in the amount of genomic change among selection regimes will reflect differences in the strength of selection pressure. We performed the Cochran-Mantel-Haenszel (CMH) test (Agresti, 2002) (popoolation2: cmh-test.pl) to identify consistent changes (relative to the base population) in allele frequencies at all polymorphic sites (*n* = 2,332,305) across the entire genome (the very small chromosome 4 was not analyzed). We note that these SNPs do not represent independent loci due to linkage. To obtain an overall indication of the strength of selection pressure in the three selection regimes, we counted the number of SNPs that had higher than an arbitrarily chosen threshold *p*-value [–log10(p) > 7] for each selection regime and chromosome separately. Using R (vs. 3.6.1) (R Core Team, 2019) we made Manhattan plots for each chromosome and selection line by plotting the negative log10-transformed *p*-values as a function of chromosome position.

### Loci Under Selection (Divergence Among Selection Lines)

Consistent differences in SNP frequencies among replicates of each pair of selection regimes (C-PF, C-UP, and PF-UF) were identified using the CMH test (popoolation2: cmh-test.pl). As such differences can be the product of both random genetic drift and selection, and the identification of loci under selection is challenging. We quantified genetic drift by performing CMH tests between the first two replicates from within each of the three thermal regimes. The resulting distribution of *p*-values across the genome is a good representation of the pattern that we expect due to drift and other sources of structure in our data. Using this distribution we selected two thresholds, more or less conservative, to detect segments of the genome where frequency changes between two selection regimes are consistent enough that we can interpret it as a product of adaptation (0.001 and 0.0001% percentile). Quantification of drift was also done using the other pairs of replicates within thermal regimes, which produced similar patterns (see Supplementary Table 1). Convergent evolution across selection regimes may occur as a consequence of both laboratory adaptation and other similarities among the three selection regimes. We quantified the pairwise overlap of significant SNPs between two selection regimes by creating 30 bins of SNP significance in one regime, and estimating the proportion of SNPs in each bin which has been under selection (according to the most conservative threshold) in another regime. In the case of independent evolution in the two regimes of a pair, we expected the proportion of SNPs in one regime to be independent of the significance level of the bins in the other regime. Alternatively, an overlap would result in an increased proportion of SNPs under selection in one regime as the significance level of bins increased (*p*-values decrease) in the other regime. We performed the same analyses with randomized *p*-values of one of the regimes as a point of reference under the null-expectation. Finally, we used the create-genewise-sync.pl script in PoPoolation2 (Kofler et al., 2011) to only analyze SNPs located in genes. This was also done for each pair of selection lines (C-PF, C-UP, and PF-UF). Similarly, we used the CMH test for each SNP independently, but averaged *p*-values across each gene. We only considered genes with more than 20 SNPs.

### Proteomic Protocol

The proteomic investigation was generally performed as described in Sørensen et al. (2017). Briefly, proteins were extracted (lysis buffer: 100 mM Triethylammonium bicarbonate (TEAB) with 0.5% sodium dodecyl sulfate (SDS) and cOmplete ULTRA Tablets (Roche Diagnostics) protease inhibitor), mechanically homogenized (Bio101, Thermo Savant FastPrep FP120 cell disruptor) on ice between cycles to keep samples cold. Protein concentration of the homogenate was determined using a Qubit^TM^ Fluorometer (Invitrogen, Life Technologies) and the Qubit Protein assay kit after centrifugation. Each sample (200 μg protein) was precipitated, dried and re-dissolved in 40 μL Dissolution Buffer with 2 μL Denaturant (iTRAQ^®^ Reagents, AB Sciex). Proteins were reduced (Reducing Reagent, iTRAQ^®^ Reagents, AB Sciex) and subsequently alkylated (Cysteine Blocking Reagent, iTRAQ^®^ Reagents, AB Sciex). Proteins were then enzymatically digested over-night to peptides using a 1:50 trypsin:protein ratio (Sequencing Grade Modified Trypsin, Promega Biotech AB). Each sample was labeled with isobaric tags for relative and absolute quantitation (iTRAQ) (Pottiez et al., 2012). Hereafter, all three samples within one replicate and a common reference were pooled for fractionation and purification. Protein samples were fractioned on columns and fractions were subsequently eluted by increasing pH. Mass spectrometry analysis was performed by high-resolution electrospray ionization tandem mass spectrometry (ESI-MS/MS) (Köcher et al., 2009). Reverse phase nanoLC separation (Dionex UltiMate RSLCnano System) was performed online coupled to the mass spectrometer (LTQ Orbitrap Velos). Three technical replicates of each pooled sample replicate were analyzed, each using a slightly different gradient to increase protein coverage. Tandem mass spectrometry parameters were: positive mode, MS scan range 300–1600 with resolution at 30,000, MS/MS fragmentation was performed using HCD (higher-energy collisional dissociation) on the 20 most intense ions with a normalized collision energy of 40, dynamic exclusion of 90 s and a minimum signal threshold of 10,000.

### Proteome Analyses

All raw data from triplicate injections of each fraction were searched and identified (Proteome Discoverer 1.4, Reporter Ions Quantifier, Percolator validator, Mascot 2.4 Search Engine and Swiss-Prot database restricted to taxonomy ‘*Drosophila’*), with the Percolator algorithm using semi-supervised machine learning and a target-decoy search strategy with reversed sequences to identify correct peptide sequence matches (Käll et al., 2007; Spivak et al., 2009). Search parameters were precursor mass tolerance 10 ppm and fragment mass tolerance 0.1 Da, maximum two missed cleavages, quantification method iTRAQ 4-plex. The strict target FDR was 0.01 for high and the relaxed target FDR was 0.05 for medium confident peptide matches. Protein quantification was based exclusively on unique peptides and among replicate experimental bias correction (Latosinska et al., 2015). The proteomics analysis identified 1319 unique protein IDs in the total data (dataset available as Supplementary Data: ‘Table 1.xlsx’). Of these, 1001 unique protein IDs were detected in at least two out of three replicate samples for all selection regimes and were retained for analysis. We only accepted proteins that were detected in two of the three samples for all regimes, to avoid a strong bias from non-detected proteins. We performed ANOVA using regimes as a categorical variable. Pairwise (*post hoc* ANOVA) comparisons between selection lines maintained in the constant, the predictably and the unpredictably fluctuating regimes were performed on the resulting dataset. All analyses were performed using the statistical software R (vs. 3.6.1) (R Core Team, 2019) (R code available upon request to the authors).

### Location of SNPs Under Selection Relative to Genes Responding Evolutionarily (Transcripts) and Plastically (Proteome) in Their Expression

We investigated whether SNPs under selection were physically linked to genes encoding proteins and transcripts responding to the thermal regimes. To visualize the locations, the genomic location of 204 transcripts that were previously found to show an evolutionary response to the fluctuating temperatures in their expression level (Manenti et al., 2018) were indicated on the Manhattan plots showing the divergence among selection lines. The genomic location of 34 loci that in this study were found to show a plastic response in protein expression after exposure to the three thermal regimes (see below) were also indicated in the Manhattan plots. To test if the overlap visualized on Manhattan plots was different from the null expectation, we estimated the observed distance from SNPs under selection to (1) genes with protein expression responding to the thermal regimes (plastic responses), and (2) genes with RNA expression responding to selection in the thermal regimes. These distances were compared to distances to a null expectation (distances to random genes). We estimated confidence intervals of the deviation from the null expectation by producing 10,000 random gene sets, each corresponding to the number of significant genes, and for each gene set estimating the average distance to nearest SNP under selection. We then subtracted these 10,000 estimates of distance to random gene sets, from the observed distance to the significant gene set. Distances between SNPs under selection and significant genes being smaller than distances between SNPs under selection and random genes would indicate linkage.

## Results

### Genomic Change (Divergence From Base Population)

Populations exposed to unpredictable fluctuations showed the largest number of consistently differentiated SNPs as compared to the base population (Table 1). This was consistent across chromosome arms, with the exception of chromosome 2L. The unpredictable fluctuations therefore likely expose the flies to a stronger selection pressure than the constant and predictably fluctuating temperatures, which both showed a lower number of consistently differentiated SNPs (Table 1). However, consistent differentiation to the base population was found among lines from all three thermal regimes suggesting that all thermal regimes imposed selection. The results of the CMH analyses were visualized as Manhattan plots for each chromosome and thermal regime (Supplementary Figure 1).

**TABLE 1:** Number of SNPs that show consistent allele frequency changes in each selection regime compared to the base population as estimated by CMH test [–log10(*p*) > 7] at the different chromosomes.

	2L	2R	3L	3R	X	Total
C	394	254	399	371	639	2057
PF	245	352	367	463	582	2009
UF	309	402	568	510	1188	2977

### Loci Under Selection (Divergence Among Selection Lines)

**FIGURE 3:**
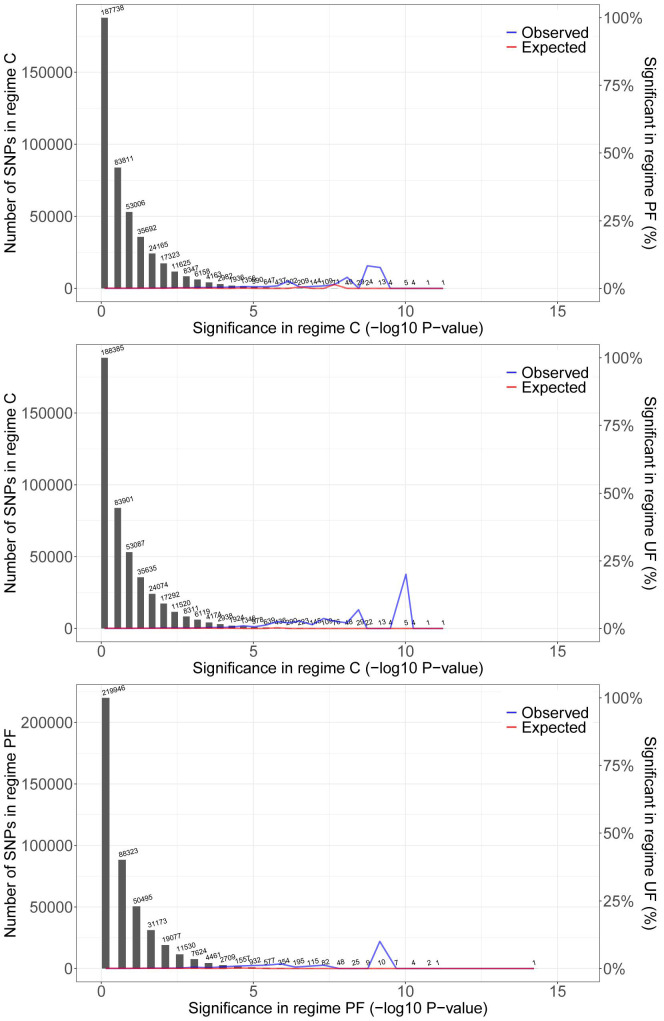
Pairwise overlap of significant SNPs among thermal regimes. Plot shows the distribution of SNP significance [–log10(*p*-values)] in one regime (gray bars), the observed proportion of SNPs that overlap with significant SNPs in the second regime, and the proportion of SNPs with randomized significance in the second regime that overlap (null-expectation). There is a consistent signal of overlap of SNPs with low *p*-values (in a small number of SNPs) in all pairwise comparisons. Thus, of the few SNPs with low *p*-values [high –log10(*p*)] in one selection regime a larger proportion than expected by chance also have low *p*-values in the other selection regimes. Only SNPs from chromosome arm 2L are presented here. Results are consistent across chromosomes (see Supplementary Figure 3).

**FIGURE 4:**
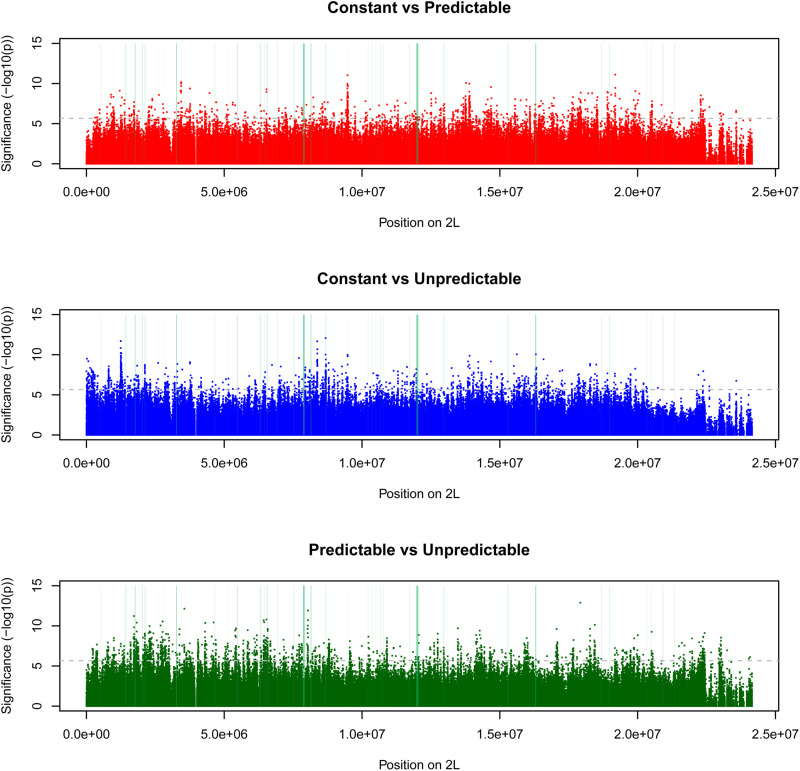
Manhattan plots comparing pairs of selection regimes. Horizontal lines indicate two thresholds used to infer patterns of differentiation due to evolutionary changes across selection regimes; 0.001 and 0.0001% percentiles. SNPs with [–log10(*p*-values)] above these thresholds can be considered evidence of divergence between selection regimes due to adaptation. Vertical lines indicate positions of loci with diverging gene expression levels (Manenti et al., 2018) (blue) or diverging protein expression levels (green), with width of line representing length of gene. Only SNPs from chromosome arm 2L are presented here. Results are consistent across chromosomes (Supplementary Figure 4).

The number of SNPs under selection according to the drift thresholds (see Supplementary Figure 2) was highest in populations exposed to the unpredictable fluctuating temperatures (Table 2). This result was consistent when selection thresholds were estimated using the other possible within-regime population pairs to quantify genetic drift, although as expected the exact threshold and therefore absolute number of SNPs inferred to be under selection varied (Supplementary Table 1). There was only a small overlap among the SNPs under selection between regimes. The magnitude of this overlap was similar in all pairwise comparisons and across all chromosomes (Figure 3: chromosome 2L, Supplementary Figure 3: all chromosomal segments). From visual inspection of the Manhattan plots comparing pairs of selection regimes (Figure 4 and Supplementary Figure 4), it is evident that the selection responses, resulting in divergence between the three selection regimes, were not limited to a single or few loci with large consistent allele frequency changes. Rather it seems that several loci, showing smaller but consistent allele frequency changes, are involved.

**TABLE 2:** Number of SNPs that show consistent allele frequency changes among thermal regimes and have a *p*-value lower than the 0.001 and 0.0001% (0.001%/0.0001%, respectively) percentile of the drift analysis at the different chromosomes and selection regimes as estimated by CMH test.

	2L	2R	3L	3R	X	Total
C vs. PF	1233/220	2895/448	1447/232	2731/548	1121/176	9427/1624
C vs. UF	1654/241	2128/306	2046/335	2677/540	2436/540	10941/1962
PF vs. UF	1426/207	2846/534	2639/493	3667/790	1972/357	12550/2381

Based on CMH analyses on SNPs located in genes, we estimated an average *p*-value per gene by averaging over all SNPs in each gene. We only analyzed genes having 20 SNPs or more. We used a similar approach to quantify drift as outlined above, but only using the 0.001% percentile. From each comparison among selection regimes, we only found a few genes having lower *p*-values than expected due to drift (Table 3). The comparisons including unpredictable fluctuations showed the highest number of genes suggested to be under selection (C-PF: 17, C-UF: 30, and PF-UF: 40). Note that 12 genes in the drift analysis have *p*-values lower than the threshold.

**TABLE 3:** Loci showing average *p*-values below what was estimated for the 99.99% drift values in the contrasts of Constant vs. Predictable fluctuating temperature (C vs. PF), Predictable fluctuating vs. Unpredictable fluctuating temperature (PF vs. UF) or Constant vs. Unpredictable fluctuating temperature (C vs. UF), respectively.

Contrast C vs. PF				
*ID*	*#SNPs*	*p*	*Gene name*	*GOterm*
FBgn0182628	98	0.0725	GD10865	GO:0016021∼integral component of membrane
FBgn0184839	29	0.0555	GD13117	GO:0050909∼sensory perception of taste, GO:0005886∼plasma membrane, GO:0016021∼integral component of membrane
FBgn0187787	26	0.0694	GD16154	
FBgn0188937	29	0.0111	GD17379	GO:0001952∼regulation of cell-matrix adhesion, GO:0051492∼regulation of stress fiber assembly
FBgn0188963	63	0.0193	GD17407	
FBgn0189060	30	0.0168	GD17510	GO:0016021∼integral component of membrane
FBgn0190058	23	0.0701	GD18535	
FBgn0193190	92	0.0754	GD21769	
FBgn0195290	62	0.0756	GD23922	GO:0003677∼DNA binding
FBgn0195511	25	0.0587	GD24156	
FBgn0195773	39	0.0575	GD24437	GO:0046872∼metal ion binding
FBgn0195794	26	0.0546	GD24459	GO:0003676∼nucleic acid binding, GO:0005524∼ATP binding
FBgn0187743	21	0.0866	Heterochromatin Protein 1D2	GO:0005634∼nucleus
FBgn0268974	58	0.0794	GD27684	
FBgn0270901	22	0.0749	GD29611	
FBgn0270917	25	0.0587	GD29627	
FBgn0268873	23	0.0438	GD27583	
**Contrast PF vs. UF**				
FBgn0187821	151	0.0587	GD16192	
FBgn0187837	47	0.0531	GD16209	GO:0006807∼nitrogen compound metabolic process, GO:0016811∼hydrolase activity, acting on carbon-nitrogen…bonds, in linear amides
FBgn0190058	22	0.0628	GD18535	
FBgn0195794	66	0.0671	GD24459	GO:0003676∼nucleic acid binding, GO:0005524∼ATP binding
FBgn0185758	27	0.0585	GD14065	
FBgn0193177	28	0.0781	GD21755	GO:0016787∼hydrolase activity
FBgn0181897	22	0.0887	GD10122	
FBgn0188378	73	0.0853	GD16791	GO:0006357∼reg. transcript. from RNA polymerase II promoter, GO:0032784∼reg. DNA-templated transcript., elongation
FBgn0188837	34	0.0513	GD17275	GO:0016021∼integral component of membrane
FBgn0188389	42	0.0484	GD16802	GO:0008270∼zinc ion binding
FBgn0187064	22	0.0268	GD15396	
FBgn0187066	86	0.0785	GD15398	GO:0016021∼integral component of membrane, GO:0008173∼RNA methyltransferase activity
FBgn0191046	27	0.0840	GD19551	GO:0008380∼RNA splicing, GO:0030532∼small nuclear ribonucleoprotein complex
FBgn0188859	22	0.0201	GD17298	GO:0004252∼serine-type endopeptidase activity
FBgn0184980	37	0.0608	GD13260	
FBgn0188002	28	0.0499	GD16383	GO:0003676∼nucleic acid binding, GO:0005524∼ATP binding, GO:0008026∼ATP-dependent helicase activity
FBgn0188033	32	0.0861	GD16416	GO:0016012∼sarcoglycan complex, GO:0016021∼integral component of membrane
FBgn0270141	26	0.0599	GD28851	GO:0006457∼protein folding, GO:0005737∼cytoplasm, GO:0005524∼ATP binding
FBgn0193396	25	0.0704	GD21981	
FBgn0184684	21	0.0864	GD12960	
FBgn0187264	36	0.0782	GD15599	GO:0016021∼integral component of membrane
FBgn0187272	51	0.0067	GD15607	
FBgn0187770	21	0.0591	GD16135	
FBgn0186916	52	0.0899	GD15248	GO:0004672∼protein kinase activity, GO:0005524∼ATP binding
FBgn0186918	99	0.0097	GD15250	GO:0000398∼mRNA splicing, via spliceosome, GO:0017070∼U6 snRNA binding, GO:0030623∼U5 snRNA binding
FBgn0184839	30	0.0753	GD13117	GO:0050909∼sensory perception of taste, GO:0005886∼plasma membrane, GO:0016021∼integral component of membrane
FBgn0188744	28	0.0729	GD17176	GO:0016491∼oxidoreductase activity
FBgn0192776	72	0.0800	GD21339	GO:0050909∼sensory perception of taste, GO:0016021∼integral component of membrane
FBgn0182763	23	0.0604	GD11003	GO:0016021∼integral component of membrane
FBgn0197138	44	0.0272	GD25858	GO:0008073∼ornithine decarboxylase inhibitor activity
FBgn0197187	31	0.0814	GD25909	GO:0004252∼serine-type endopeptidase activity
FBgn0188910	32	0.0751	GD17350	GO:0005634∼nucleus
FBgn0188937	24	0.0425	GD17379	GO:0030335∼positive regulation of cell migration, GO:0016021∼integral component of membrane
FBgn0187661	21	0.0757	GD16020	
FBgn0195562	25	0.0487	GD24212	GO:0016787∼hydrolase activity
FBgn0194793	38	0.0796	GD23408	
FBgn0270901	22	0.0810	GD29611	
FBgn0268296	21	0.0789	GD27006	
FBgn0270617	26	0.0674	GD29327	
FBgn0270329	55	0.0831	GD29039	
**Contrast C vs. UF**				
FBgn0187885	44	0.0877	GD16258	
FBgn0195794	67	0.0471	GD24459	GO:0003676∼nucleic acid binding, GO:0005524∼ATP binding
FBgn0184467	23	0.0631	GD12740	GO:0016021∼integral component of membrane, GO:0004252∼serine-type endopeptidase activity
FBgn0188360	28	0.0320	GD16772	GO:0016021∼integral component of membrane, GO:0004252∼serine-type endopeptidase activity
FBgn0188361	49	0.0320	GD16773	GO:0016021∼integral component of membrane, GO:0004252∼serine-type endopeptidase activity
FBgn0261743	46	0.0864	Desaturase 2	GO:0006633∼fatty acid biosynthetic process, GO:0016021∼integral component of membrane
FBgn0188033	34	0.0037	GD16416	GO:0016012∼sarcoglycan complex, GO:0016021∼integral component of membrane
FBgn0270141	30	0.0750	GD28851	GO:0006457∼protein folding, GO:0005737∼cytoplasm, GO:0005524∼ATP binding
FBgn0194698	29	0.0859	GD23312	GO:0006351∼transcription, DNA-templated, GO:0003899∼DNA-directed RNA polymerase activity
FBgn0196911	24	0.0324	GD25625	GO:0051539∼4 iron, 4 sulfur cluster binding
FBgn0187272	50	0.0898	GD15607	
FBgn0187291	53	0.0644	GD15627	
FBgn0188770	26	0.0878	GD17206	GO:0008270∼zinc ion binding
FBgn0182763	21	0.0234	GD11003	GO:0016021∼integral component of membrane
FBgn0197138	30	0.0618	GD25858	GO:0008073∼ornithine decarboxylase inhibitor activity
FBgn0188402	25	0.0365	GD16815	GO:0006744∼ubiquinone biosynthetic process, GO:0055114∼oxidation-reduction process
FBgn0186312	37	0.0725	GD14632	GO:0002949∼tRNA threonylcarbamoyladenosine modification
FBgn0195553	103	0.0865	GD24203	GO:0016021∼integral component of membrane, GO:0016791∼phosphatase activity
FBgn0195555	42	0.0421	GD24205	GO:0072669∼tRNA-splicing ligase complex, GO:0046872∼metal ion binding
FBgn0187310	43	0.0550	GD15648	GO:0001700∼embryonic development via the syncytial blastoderm, GO:0007259∼JAK-STAT cascade, GO:0005622∼intracellular
FBgn0184291	24	0.0721	GD12564	GO:0006886∼intracellular protein transport, GO:0006913∼nucleocytoplasmic transport, GO:0007264∼small GTPase mediated signal transduction
FBgn0193948	80	0.0771	GD22547	GO:0006464∼cellular protein modification process
FBgn0270798	21	0.0480	GD29508	
FBgn0268292	25	0.0457	GD27002	
FBgn0268296	21	0.0473	GD27006	
FBgn0268783	39	0.0615	GD27493	
FBgn0268450	33	0.0818	GD27160	
FBgn0268614	47	0.0401	GD27324	
FBgn0268873	28	0.0448	GD27583	
FBgn0269334	32	0.0309	GD28044	

*Only loci with at least 20 SNPs were included. The FlyBase accession ID, number of SNPs for each locus (#SNPs), the average p-value (p), Gene name and associated functional annotation (GOterm) are given in the table. Gene name and GOterms (truncated for some redundant terms) were obtained by the Functional Annotation Table of DAVID (The Database for Annotation, Visualization and Integrated Discovery v6.8) (Huang et al., 2009) and FlyBase (flybase.org).*

The functional annotation of the identified genes was only available for about half the genes (Table 3). Attempting to identify likely *D. melanogaster* orthologs resulted in ‘uncharacterized proteins.’ Among the characterized genes, many were involved in regulation of expression of the genome, i.e., processes related to transcription, translation and post-transcriptional regulation or regulation (e.g., mRNA, splicing, tRNAs, chaperonins involved in protein folding). Additionally, the desaturase gene Desat 2, involved in the modifications of fatty acids, was identified.

### Plastic Proteomic Responses

Of the 1001 analyzed proteins, 34 proteins showed differences in their expression levels among the three thermal regimes. *Post hoc* pairwise comparisons showed that none of the proteins were differentially expressed among all three regimes. Most of protein expression differences were found between the unpredictably fluctuating as compared to either the constant (24 proteins) or the predictably fluctuating (14 proteins) regime, respectively (Table 4). Eight of these proteins were shared between the two contrasts and thus showed unique expression in the unpredictable regime. The proteins showing differential expression among flies exposed to the different thermal regimes covered a range of biological functions. Most notable are proteins known to respond to environmental cues, including several heat shock proteins (Hsp60, Hsc70-5, and Hsp83), turandot proteins (Turandot A, Turandot X), a metallothionein and two odorant-binding proteins. We also found several signaling proteins (Calmodulin, Pellino) as well as proteins involved in the transcriptional and translational machinery (ribosomal proteins, elongation and translation initiation factors) and turnover (Ubiquitin-conjugating enzymes).

**TABLE 4:** Proteomics results.

Accession	Description	Contrast	PF-C	Contrast	UF-C	Contrast	UF-PF
		
		FC	P	FC	P	FC	P
P48375	12 kDa FK506-binding protein	1.01	0.91	1.10	***0.03***	1.08	***0.05***
O02649	60 kDa heat shock protein, mitochondrial	1.05	0.14	1.13	***0.00***	1.07	***0.04***
Q9VA91	40S ribosomal protein S7	0.97	***0.03***	1.00	0.96	1.03	***0.04***
B4IL76	40S ribosomal protein S3	0.96	0.30	0.92	***0.03***	0.96	0.26
B4II57	Protein Turandot C	1.12	0.67	0.49	***0.02***	0.43	***0.01***
P35128	Ubiquitin-conjugating enzyme E2 N	1.03	0.86	1.16	***0.03***	1.14	0.06
P29845	Heat shock 70 kDa protein cognate 5	1.05	0.32	1.11	***0.03***	1.06	0.18
B4II58	Protein Turandot A1/2	1.28	0.49	0.47	0.13	0.37	***0.03***
P02828	Heat shock protein 83	1.03	0.24	1.09	***0.01***	1.06	***0.04***
P62152	Calmodulin	0.63	***0.05***	0.60	***0.04***	0.95	0.96
P13060	Elongation factor 2	0.97	0.25	0.93	***0.03***	0.97	0.24
Q8I1F4	rRNA 2’-O-methyltransferase fibrillarin	0.92	***0.01***	0.96	0.10	1.04	0.15
Q8MMC4	Protein CDV3 homolog	0.81	0.15	0.68	***0.05***	0.84	0.33
Q24046	Sodium/potassium-transporting ATPase subunit beta-1	0.98	0.34	1.03	0.25	1.05	***0.04***
Q24388	Larval serum protein 2	1.22	0.28	1.42	***0.04***	1.17	0.32
Q8SY61	General odorant-binding protein 56d	1.21	0.08	1.27	***0.03***	1.05	0.75
P07182	Chorion protein S36	0.97	0.82	0.77	***0.01***	0.80	***0.03***
Q9VAI6	General odorant-binding protein 99b	1.23	0.14	1.78	***0.00***	1.44	***0.00***
P24511	Chorion protein S16	1.01	0.98	0.84	0.06	0.84	***0.05***
Q9V7N5	V-type proton ATPase subunit C	0.95	0.22	0.91	***0.03***	0.96	0.35
P04357	Metallothionein-1	1.09	0.77	1.44	***0.03***	1.33	0.07
P48598	Eukaryotic translation initiation factor 4E	1.04	0.42	0.94	0.17	0.90	***0.03***
P41073	Zinc finger protein on ecdysone puffs	0.95	0.19	0.92	***0.04***	0.97	0.50
Q9VPH7	Eukaryotic peptide chain release factor subunit 1	1.18	***0.05***	1.04	0.77	0.88	0.12
Q7KML2	Probable peroxisomal acyl-coenzyme A oxidase 1	1.11	***0.05***	1.09	0.08	0.99	0.94
Q27237	Protein tumorous imaginal disks, mitochondrial	1.15	***0.02***	1.14	***0.03***	0.99	0.97
O77237	Protein pellino	0.76	***0.00***	0.78	***0.01***	1.03	0.90
P16163	Uricase	1.02	0.99	0.53	***0.01***	0.52	***0.01***
Q9VAI1	Probable complex I intermediate-associated protein 30, mitochondrial	0.82	***0.02***	0.80	***0.02***	0.98	0.88
Q9VTY6	Ubiquitin-conjugating enzyme E2 C	1.19	***0.02***	1.10	0.21	0.92	0.22
Q9VVW8	ATP-dependent (S)-NAD(P)H-hydrate dehydratase	1.13	0.29	1.26	***0.04***	1.11	0.33
P42787	Carboxypeptidase D	1.12	***0.01***	1.01	0.86	0.91	***0.02***
O02437	Protein yellow	0.99	0.97	0.90	***0.04***	0.91	0.06
Q9I7T7	La-related protein CG11505	0.94	0.11	0.85	***0.01***	0.91	***0.05***

*UniProt accession ID and protein name for Fold change (log2 FC) and p-value for each pairwise contrast is given. P-values in italics and bold are considered as significant (p < 0.05).*

### SNPs Under Selection Are Not Closer to Genes That Show Expression Responses (Evolutionary or Plastic) Than Random Genes

**FIGURE 5:**
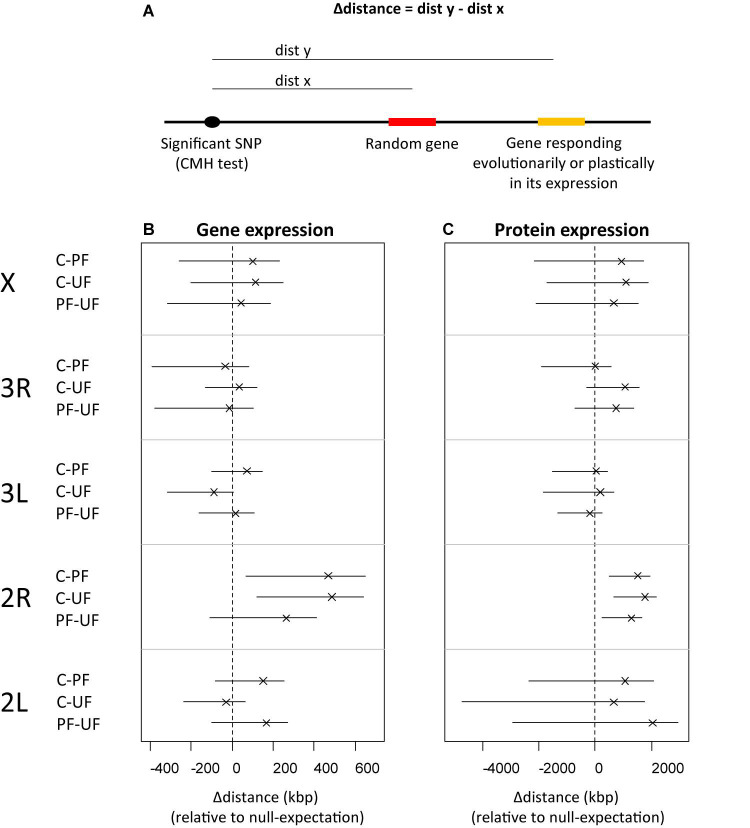
Represents an analysis of distance between SNPs detected to be under selection in the thermal regimes detected by the CMH test and genes that showed an evolutionary response in their gene expression and genes that show a plastic response in their proteome expression. **(A)** illustration of the metric used: Δdistance (the average distance between SNPs under selection and nearest gene showing expression response – the average distance between SNPs under selection and nearest gene out of a random set of 204 genes in gene expression and 34 genes in protein expression). **(B,C)** Δdistance calculated for each chromosome and thermal regime comparison. Crosses (x) are medians and horizontal lines (–) are 95% confidence intervals both based on 10000 random sets of genes. **(B)** Shows results of genes that show an evolutionary response in gene expression and **(C)** the results of genes that show a plastic response in proteome expression.

The loci that have shown evolutionary responses in their level of transcription (Manenti et al., 2018) or proteome expression (this study) did not show obvious signs of physical linkage to SNPs under selection in the three thermal regimes. The average distance from these SNPs to nearest genes showing expression responses were in no comparison shorter than their average distance to random genes (Figure 5) suggesting that (1) evolutionary responses in gene expression to the thermal regimes are not caused by selection on cis-elements, and (2) genes that respond plastically to the thermal regimes were not under strong selection.

## Discussion

In this study, we applied thermal regimes with temperature variation on replicated populations of *D. simulans* to mimic naturally occurring daily variation (Kingsolver et al., 2009; Bozinovic et al., 2011; Manenti et al., 2014). We found a signal of the 20 generations of experimental evolution as flies maintained in constant, predictable or unpredictable fluctuating environments showed independent genomic differentiation. Natural populations are exposed, and likely adapted, to thermal fluctuations. Thus, as temperatures in natural habitats are not predictable, the unpredictable regime could be argued to represent the most natural condition (Colinet et al., 2015). Our genomic data suggests that the selection pressure in the unpredictable fluctuating regime is distinct from the constant and predictable fluctuating thermal environments. This result corroborates the induced response (i.e., results from the proteomic analysis presented here) and results from an earlier study where the transcriptome responses of the same selection lines showed similar patterns of a stronger response to unpredictable temperature fluctuations (Manenti et al., 2018). Furthermore, previous studies have documented that exposure to unpredictable temperature fluctuation was more stressful compared to a predictable fluctuating temperature regime with the same mean temperature (as measured by decreased performance in several life history and stress tolerance traits) (Manenti et al., 2014), and that performance of the lines selected in the unpredictable fluctuating regime had evolved enhanced stress tolerance (Manenti et al., 2015).

The finding that the unpredictable fluctuations impose a distinct selection pressure (even if the amplitude was smaller than that of the predictable fluctuating regime) suggests that the unpredictable selection regime does not resemble natural thermal profiles. If our unpredictably fluctuating regime did represent the natural environment, it could be hypothesized to represent the least novel environment and show the least change from the base population. It is often hypothesized that keeping up with environmental change by induction of plastic responses in variable environments can be costly (Dewitt et al., 1998; for an empirical example, see Kristensen et al., 2008). In this study (and in Manenti et al., 2018), predictability rather than amplitude seems to impose the strongest effect on both plastic and evolutionary responses. This points to that the cost of thermal fluctuations (and maybe plasticity generally) is related to the costs of evaluating the environment rather than the amount of regulation needed (Dewitt et al., 1998).

### Distinct Selection Responses Among Thermal Regimes

We looked for SNPs that show similar selective responses across selection regimes by comparing *p*-values of each SNP between each pair of the three selection regimes, and for each chromosome separately (Figure 3 and Supplementary Figure 3). There was not a strong pattern that SNPs with low *p*-values [high –log10(p)] in one selection regime also had low *p*-values in the other selection regimes, suggesting that in general the loci under selection are unique to each selection regime. We do note, however, that we did identify an overlap in a small number of SNPs across pairwise comparisons of the three selection regimes. This could represent a weak signal of a common evolutionary response to thermal regimes or shared laboratory conditions, not including temperature, that affect the same loci. While laboratory adaptation can be prominent, for thermal tolerance it does not seem to be a general concern (Maclean et al., 2018), and in this study did not overshadow the effect of the individual regimes.

Our findings support the conclusion that independent selection has occurred in response to the three different selection regimes. This underlines that fluctuating thermal regimes are affecting organism in a very complex way. Thus, the effect of natural fluctuations can be proposed to be associated both with amplitude (e.g., Terblanche et al., 2010; Bozinovic et al., 2011; Ma et al., 2015), but independently also by a marked effect of predictability, possibly mediated by the cost of continuously evaluating and adjusting to the present conditions (Dewitt et al., 1998).

### Proteomic Response to Thermal Fluctuation Regimes

Among the significantly differentially expressed proteins several heat shock and Turandot genes were found. Qualitative comparison to the study of the evolved transcriptome by Manenti et al. (2018) suggests a substantial overlap in functional groups, e.g., ribosomal genes, ubiquitin conjugating enzymes, Turandot genes, odorant binding proteins, but surprisingly no overlap with the heat shock genes found in this study. The Turandot and heat shock genes expressed in flies exposed to fluctuations (and particular to unpredictable fluctuations) indicate a heat stress response (Ekengren and Hultmark, 2001; Sørensen et al., 2003). The modest expression levels and a previous transcriptomic study suggest that the up-regulation due to temperature fluctuations are mainly constitutive, rather than tracking the temperature fluctuations (Manenti et al., 2018). The three heat shock proteins found here (Hsp83, Hsc70-5, and Hsp60) support this notion, as they have relatively high constitutive expression and are not expressed at much higher levels by stress (Feder and Hofmann, 1999; Sørensen et al., 2005). The other proteins detected as differentially expressed have no known connection to thermal tolerance, but include proteins important for efficient transcription and translation which might serve to maintain functioning of the cellular machinery allowing animals to survive and reproduce. The low number of proteins found to be differentially expressed is likely due to the relative benign extreme temperatures and the resulting low fold-change induced, but the low number of differentially expressed proteins does also indicate that regulation of quite few proteins is adequate for maintaining cellular function at variable temperatures. A recent study compared heat tolerance and the transcriptomic response to developmental acclimation at different mean temperatures (15 or 25°C), and either fluctuating or constant temperatures (Sørensen et al., 2016b). They found that fluctuations affected heat tolerance markedly, despite of a low number of transcripts (a few hundred) being differentially expressed between the constant and fluctuating regimes differentially expressed. In contrast, a large number of transcripts responded differences in mean temperatures (6000–8000 different expressed) (Sørensen et al., 2016b). This suggests that while some stress response proteins were induced by fluctuations, the molecular underpinnings of benign fluctuations might differ substantially from the pathways known to be involved with more extreme thermal acclimation or hardening.

### Evolutionary Adaptation to Fluctuating Temperatures

The loci affected by selection were (for those that could be functionally annotated) related to regulation of the expression of the genome, rather than to genes with known functions in thermal responses or tolerance. This suggests that the evolutionary adaption to fluctuations within benign temperatures is to a large extent achieved by trans-regulation of the genome, rather than by increasing thermal stability and adaptation of proteins seen in extremophiles (Jollivet et al., 2012).

The distribution of distances between SNPs seemingly under selection and the transcriptome differences imposed by selection by the thermal regimes also showed no sign of cis-regulation (i.e., short distances). For the transcripts showing a change in expression following selection this might indicate some degree of trans-regulation. For the proteins responding to our thermal regimes this might suggest that evolved differences and the plastic response are not generally achieved by common genes. However, we acknowledge that linkage and linkage disequilibrium might contribute to our results. Unfortunately, lack of information on linkage in *D. simulans* and the pooled sequencing data prevents us from a more dedicated analysis of this effect. One potential cis-regulated mechanism is related to the Desat2 gene (a lipid desaturase), which could be related to membrane homeoviscous adaptation (Hazel, 1995). However, this remains a hypothesis for future testing.

Fluctuating temperature regimes can vary in terms of period, amplitude, rate of temperature change and predictability in addition to the mean temperature. Thus, even if studies with thermal fluctuations can be considered more ecologically relevant than those with constant temperatures, it is by no means simple to compare results across laboratory studies and to extrapolate findings to field conditions (Ketola and Kristensen, 2017). We found no marked overlap between loci being affected by selection and the proteins induced by the regimes suggesting that the evolutionary and plastic responses are achieved by distinct genes. The genes associated by many significant SNPs suggest that transcription, translation and post-translational modification are targets of evolutionary change. Increased expression of Turandot proteins under fluctuations support this group of genes/proteins as emerging candidates for mediating thermal acclimation to fluctuations. Based on our findings we argue that it is important to acknowledge that predictable and unpredictable (and constant) thermal environments have different impacts on fitness. While other studies have shown that constant and fluctuating temperatures induce different plastic responses and evolutionary pressures (Botero et al., 2015; Dey et al., 2016), this aspect is often ignored in functional and evolutionary studies on thermal adaptation. This is critical because climate models predict more variable and less predictable climates in the future (IPCC, 2014).

## Data Availability Statement

The raw genome sequencing data was uploaded to the NCBI SRA (short read archive) BioProject accession: PRJNA629467.

## Author Contributions

JS and VL acquired the financial support for the project leading to this publication. JS, TM, and VL conceived and designed the study. JS and TM performed experiments and data collection. JS, JB, and MS analyzed the data and wrote first draft of the manuscript. All authors contributed to the final draft and approved the final version of the manuscript.

## Conflict of Interest

The authors declare that the research was conducted in the absence of any commercial or financial relationships that could be construed as a potential conflict of interest.

## Footnotes

1 www.dmi.dk

2 https://github.com/FelixKrueger/TrimGalore

3 https://ensembl.org
