# Supplementary material for: Pronounced Plastic and Evolutionary Responses to Unpredictable Thermal Fluctuations in Drosophila simulans
Source: Front Genet. 2020 Oct 28;11:555843. doi: 10.3389/fgene.2020.555843 (PMC7655653; doi:10.3389/fgene.2020.555843)
Supplement: Supplementary file 2 [file Data_Sheet_1.pdf]

Supplementary material for

Submission for the Research Topic: Coping with Climate Change: A Genomic Perspective on Thermal Adaptation to Frontiers in Genetics

Jesper G Sørensen[1], Tommaso Manenti[1,2], Jesper S Bechsgaard[1], Mads F Schou[3], Torsten N Kristensen[4], Volker Loeschcke[1]

Pronounced plastic and evolutionary responses to unpredictable thermal fluctuations in *Drosophila simulans*

[1] Department of Biology, Aarhus University, Ny Munkegade 116, Bldg. 1540, 8000 Aarhus C, Denmark

[2] Present address: Laboratori Biokyma srl, Loc.Mocaia 44b, 52031 Anghiari, AR, Italy

[3] Department of Biology, Lund University, Sölvegatan 37, Lund, Sweden

[4] Department of Chemistry and Bioscience, Aalborg University, Frederik Bajers Vej 7H, Aalborg, DK 9220, Denmark

Corresponding author: Jesper G Sørensen (jesper.soerensen@bio.au.dk)

Running title: Adaptive responses to thermal fluctuations

Keywords: heat tolerance, thermal fluctuations, genomics, proteomics, *Drosophila simulans*

Supplemental table 1. Number of SNPs that show consistent allele frequency changes and have a p-value lower than the threshold percentile (0.001% and 0.0001%, respectively) of the drift analysis at the different chromosomes and selection regimes as estimated by CHM test. L1, L2 and L3 represent independent selection lines for each regime. The –log10(p)) value corresponding to the two thresholds are given in the legends of Figures S2a-e.

		L1 vs L2		*L1 vs L3*		*L2 vs L3*	
		0.001	*0.0001*	*0.001*	*0.0001*	*0.001*	*0.0001*
C vs PF	*2L*	*1233*	*220*	*1486*	*267*	*3374*	*740*
C vs UF	*2L*	*1654*	*241*	*1980*	*320*	*4466*	*911*
PF vs UF	*2L*	*1426*	*207*	*1709*	*288*	*3918*	*798*
C vs PF	*2R*	*2895*	*448*	*3326*	*810*	*4605*	*1127*
C vs UF	*2R*	*2128*	*306*	*2505*	*526*	*3635*	*763*
PF vs UF	*2R*	*2846*	*534*	*3244*	*878*	*4470*	*1173*
C vs PF	*3L*	*1447*	*232*	*1813*	*238*	*2547*	*537*
C vs UF	*3L*	*2046*	*335*	*2480*	*348*	*3295*	*760*
PF vs UF	*3L*	*2639*	*493*	*3162*	*516*	*4158*	*1091*
C vs PF	*3R*	*2731*	*548*	*4062*	*705*	*3933*	*846*
C vs UF	*3R*	*2677*	*540*	*3950*	*700*	*3815*	*839*
PF vs UF	*3R*	*3667*	*790*	*5422*	*1008*	*5257*	*1193*
C vs PF	*X*	*1121*	*176*	*1197*	*196*	*1533*	*296*
C vs UF	*X*	*2436*	*540*	*2571*	*588*	*3256*	*790*
PF vs UF	*X*	*1972*	*357*	*2108*	*399*	*2665*	*545*

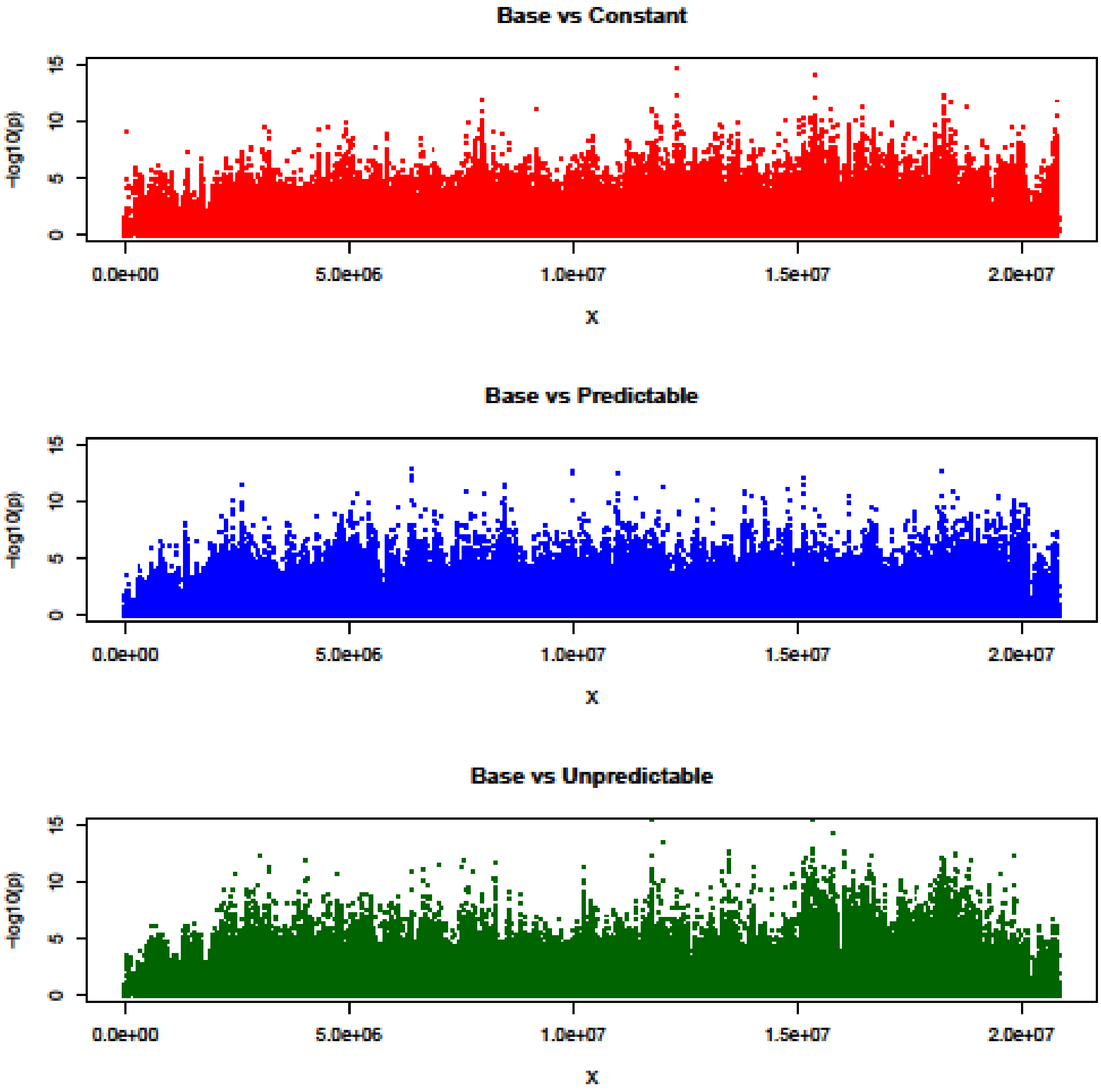

Supplemental figure 1a. Manhattan plots showing the –log10(p-value) of SNPs obtained by CHM tests as a function of genomic position (here chromosome X). The three lines exposed to 20 generations of laboratory natural selection in the thermal regimes: Constant (red), Predictable fluctuating (Blue) and Unpredictable fluctuating (Green) were compared to the freshly field collected population (Base). X-axis represent position on chromosome.

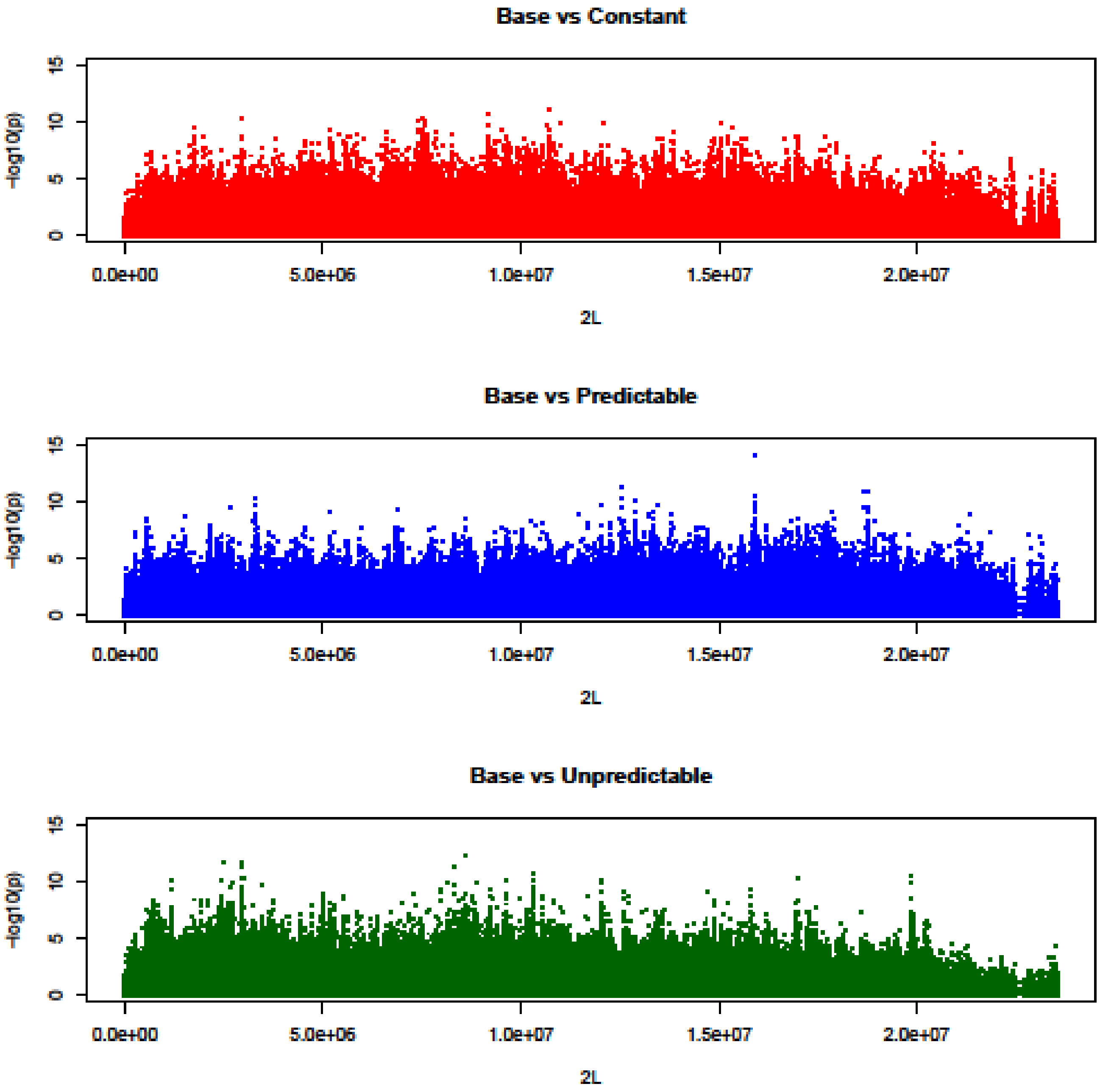

Supplemental figure 1b. Manhattan plots showing the –log10(p-value) of SNPs obtained by CHM tests as a function of genomic position (here chromosome 2L). The three lines exposed to 20 generations of laboratory natural selection in the thermal regimes: Constant (red), Predictable fluctuating (Blue) and Unpredictable fluctuating (Green) were compared to the freshly field collected population (Base). X-axis represent position on chromosome.

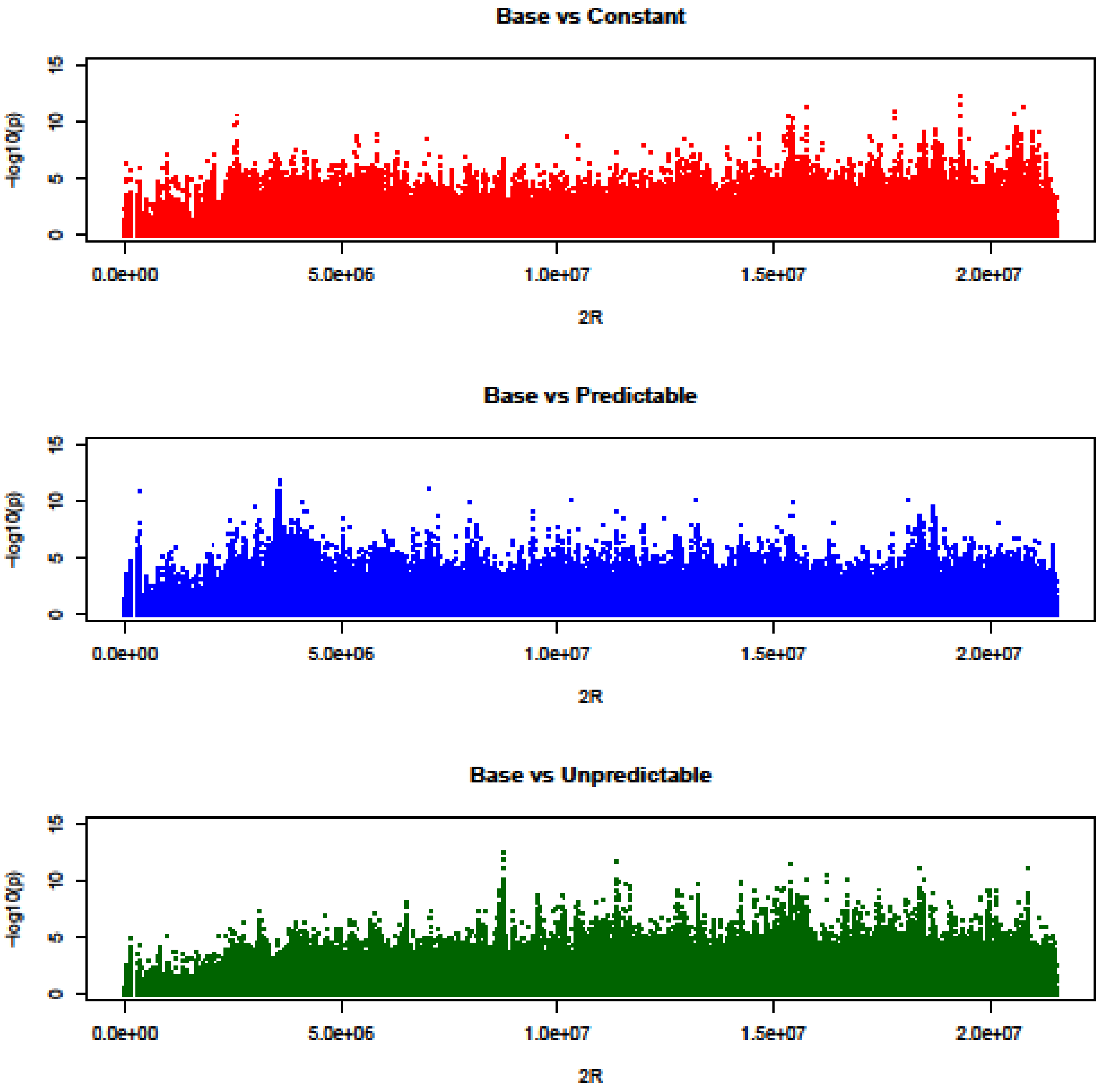

Supplemental figure 1c. Manhattan plots showing the –log10(p-value) of SNPs obtained by CHM tests as a function of genomic position (here chromosome 2R). The three lines exposed to 20 generations of laboratory natural selection in the thermal regimes: Constant (red), Predictable fluctuating (Blue) and Unpredictable fluctuating (Green) were compared to the freshly field collected population (Base). X-axis represent position on chromosome.

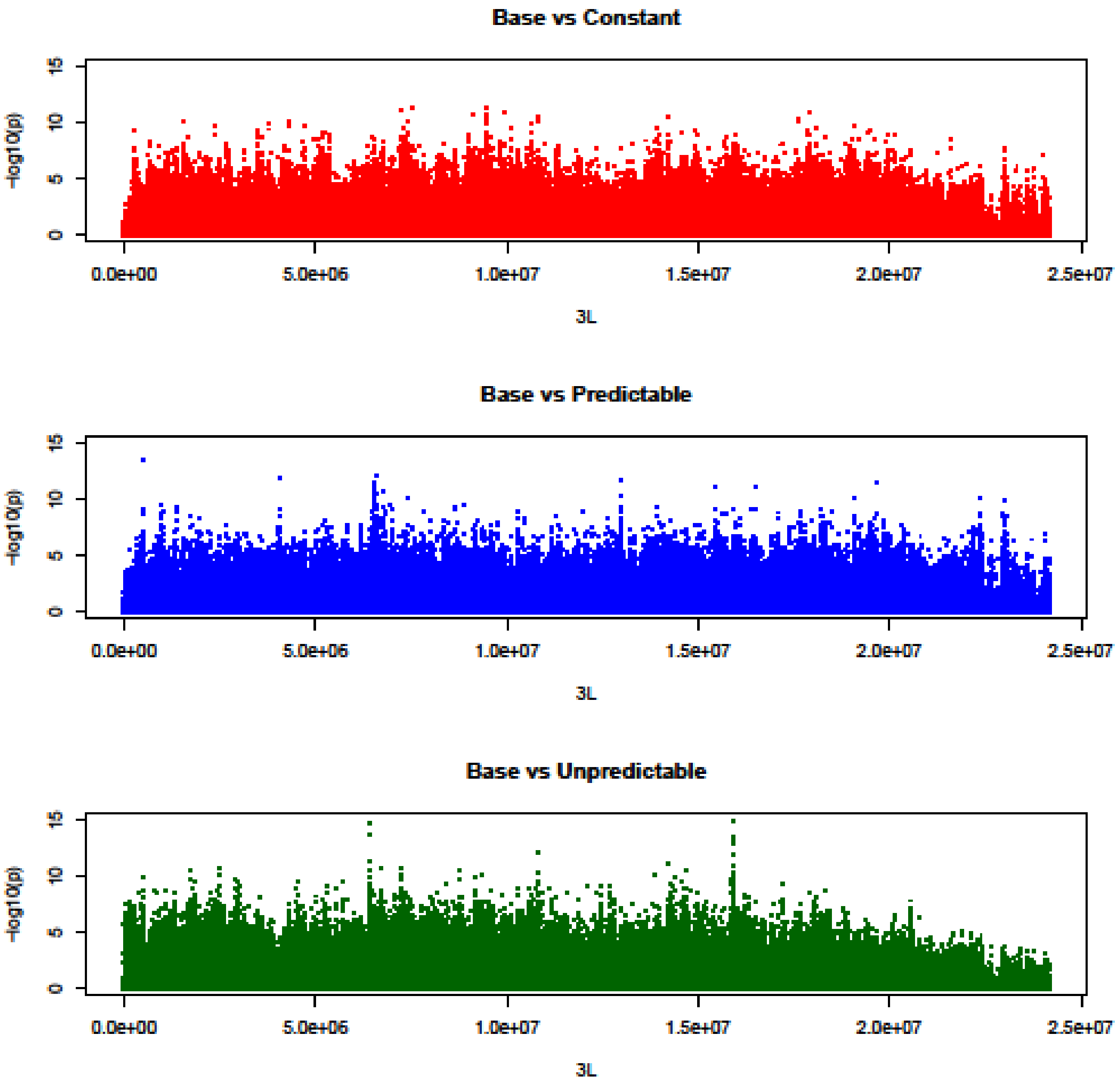

Supplemental figure 1d. Manhattan plots showing the –log10(p-value) of SNPs obtained by CHM tests as a function of genomic position (here chromosome 3L). The three lines exposed to 20 generations of laboratory natural selection in the thermal regimes: Constant (red), Predictable fluctuating (Blue) and Unpredictable fluctuating (Green) were compared to the freshly field collected population (Base). X-axis represent position on chromosome.

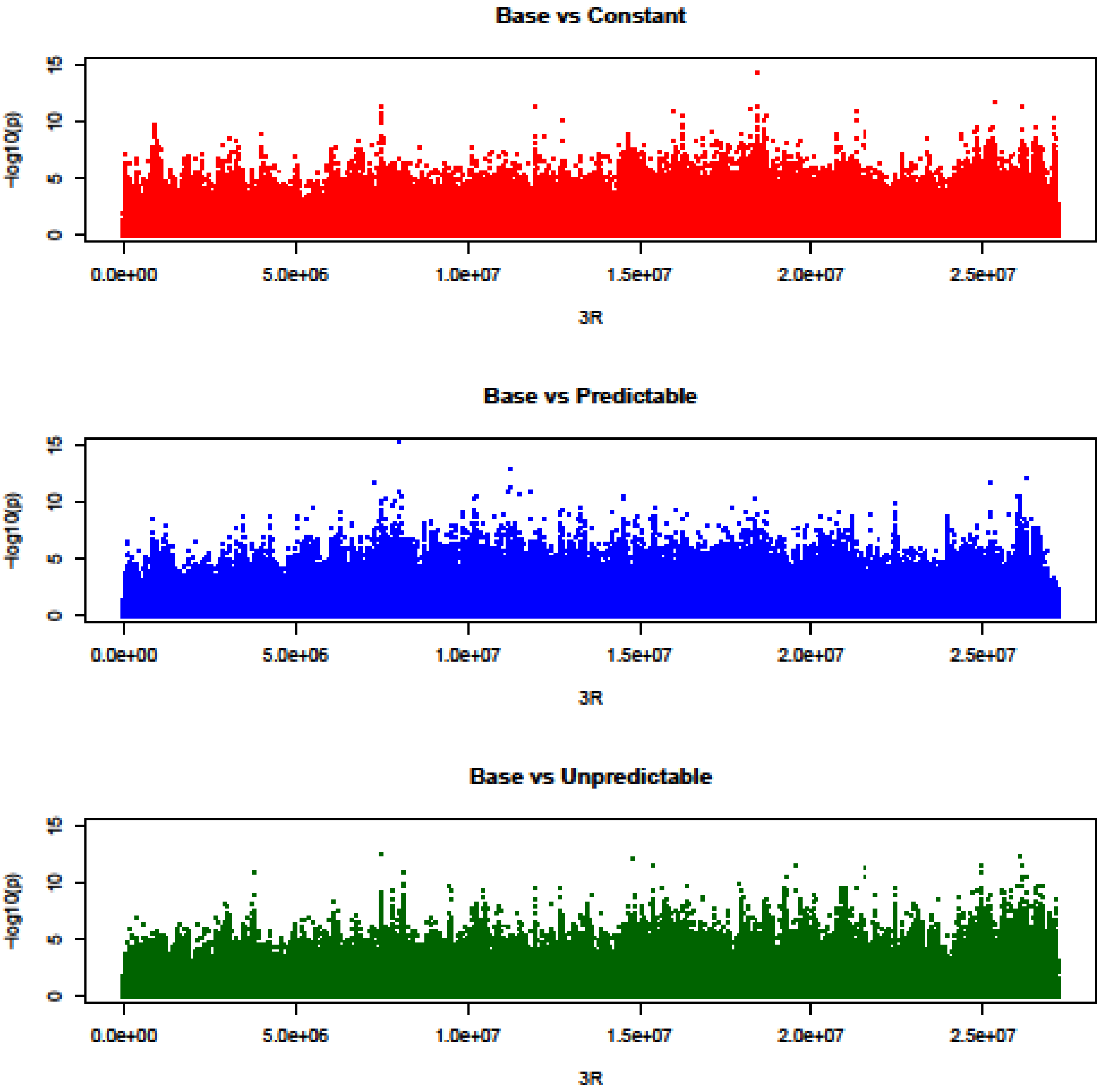

Supplemental figure 1e. Manhattan plots showing the –log10(p-value) of SNPs obtained by CHM tests as a function of genomic position (here chromosome 3R). The three lines exposed to 20 generations of laboratory natural selection in the thermal regimes: Constant (red), Predictable fluctuating (Blue) and Unpredictable fluctuating (Green) were compared to the freshly field collected population (Base). X-axis represent position on chromosome.

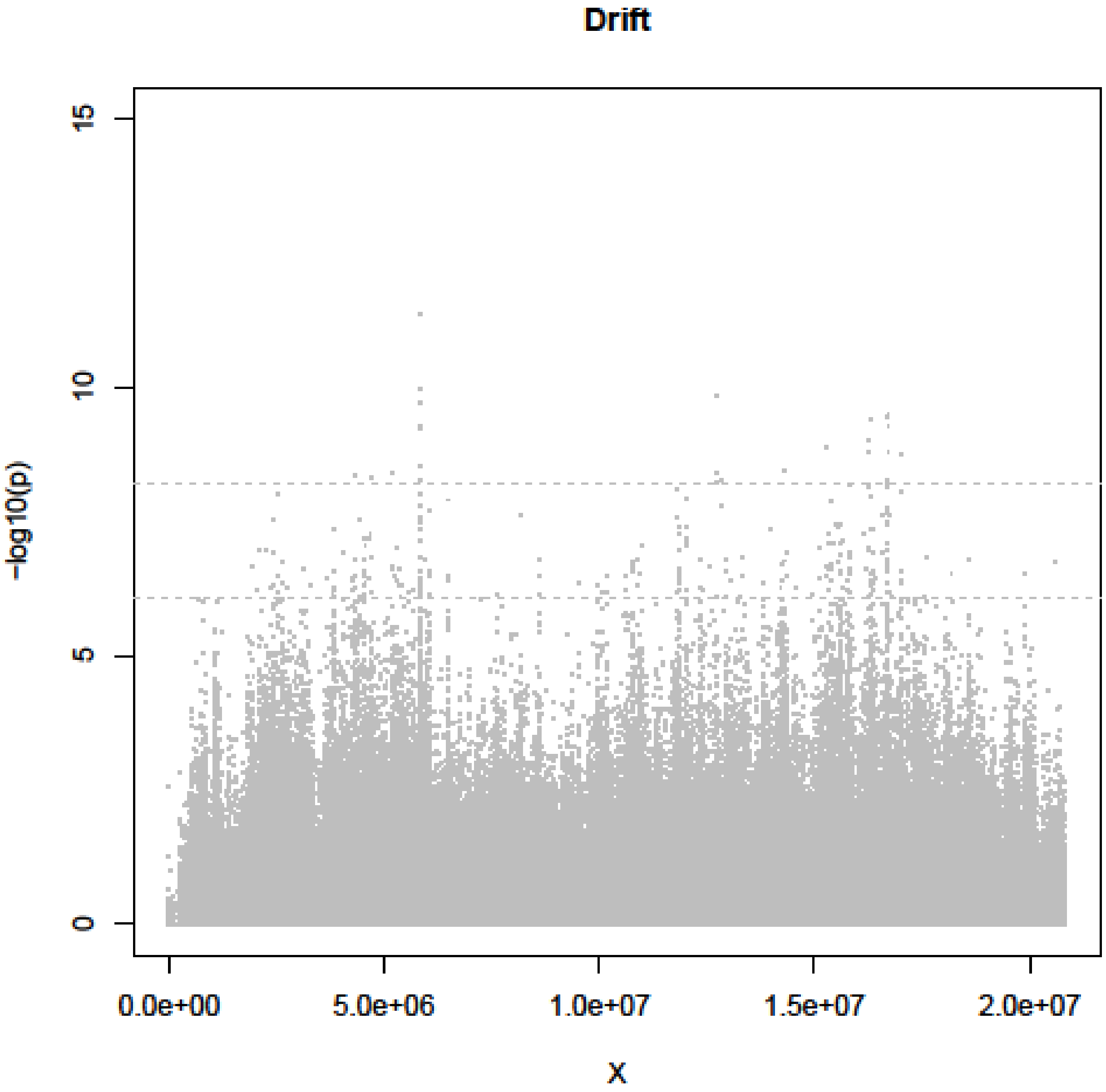

Supplemental figure 2a. We quantified genetic drift by performing CMH tests between replicate 1 and 2 from each of the three thermal regimes. The resulting distribution of p-values across the genome is a good representation of the pattern that we may expect due to drift and other sources of structure in our data. Using this distribution we determined two cut-offs used to detect segments of the genome where frequency changes between two selection regimes are consistent enough that we can interpret it as a product of selection The 0.001% and 0.0001% percentile corresponded to -log10(p) thresholds of 6.06 and 8.20, respectively. Each chromosomal segment is shown separately (here chromosome X). X-axis represent position on chromosome.

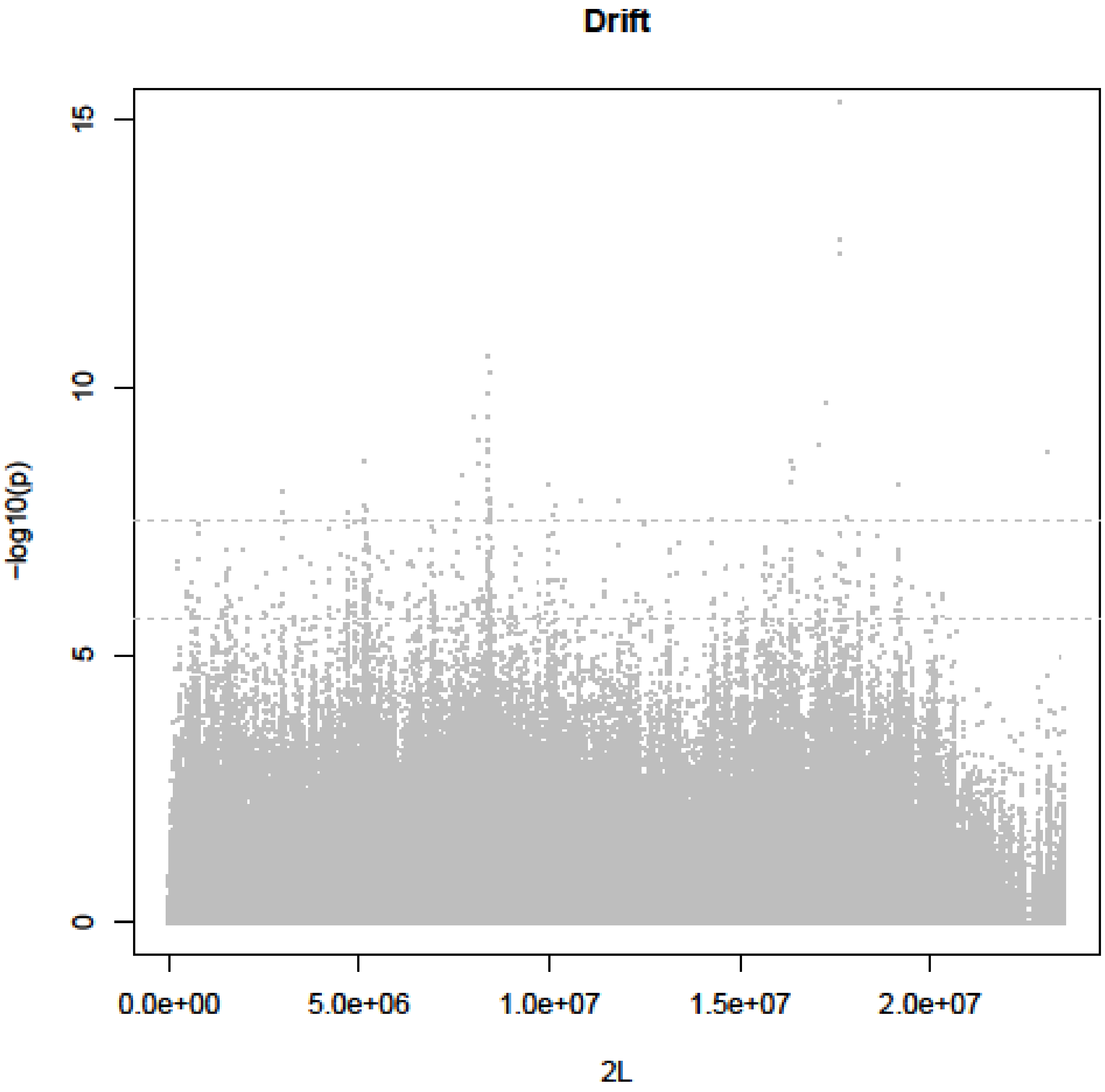

Supplemental figure 2b. We quantified genetic drift by performing CMH tests between replicate 1 and 2 from each of the three thermal regimes. The resulting distribution of p-values across the genome is a good representation of the pattern that we may expect due to drift and other sources of structure in our data. Using this distribution we determined two cut-offs used to detect segments of the genome where frequency changes between two selection regimes are consistent enough that we can interpret it as a product of selection. The 0.001% and 0.0001% percentile corresponded to -log10(p) thresholds of 5.66 and 7.50, respectively. Each chromosomal segment is shown separately (here chromosome 2L). X-axis represent position on chromosome.

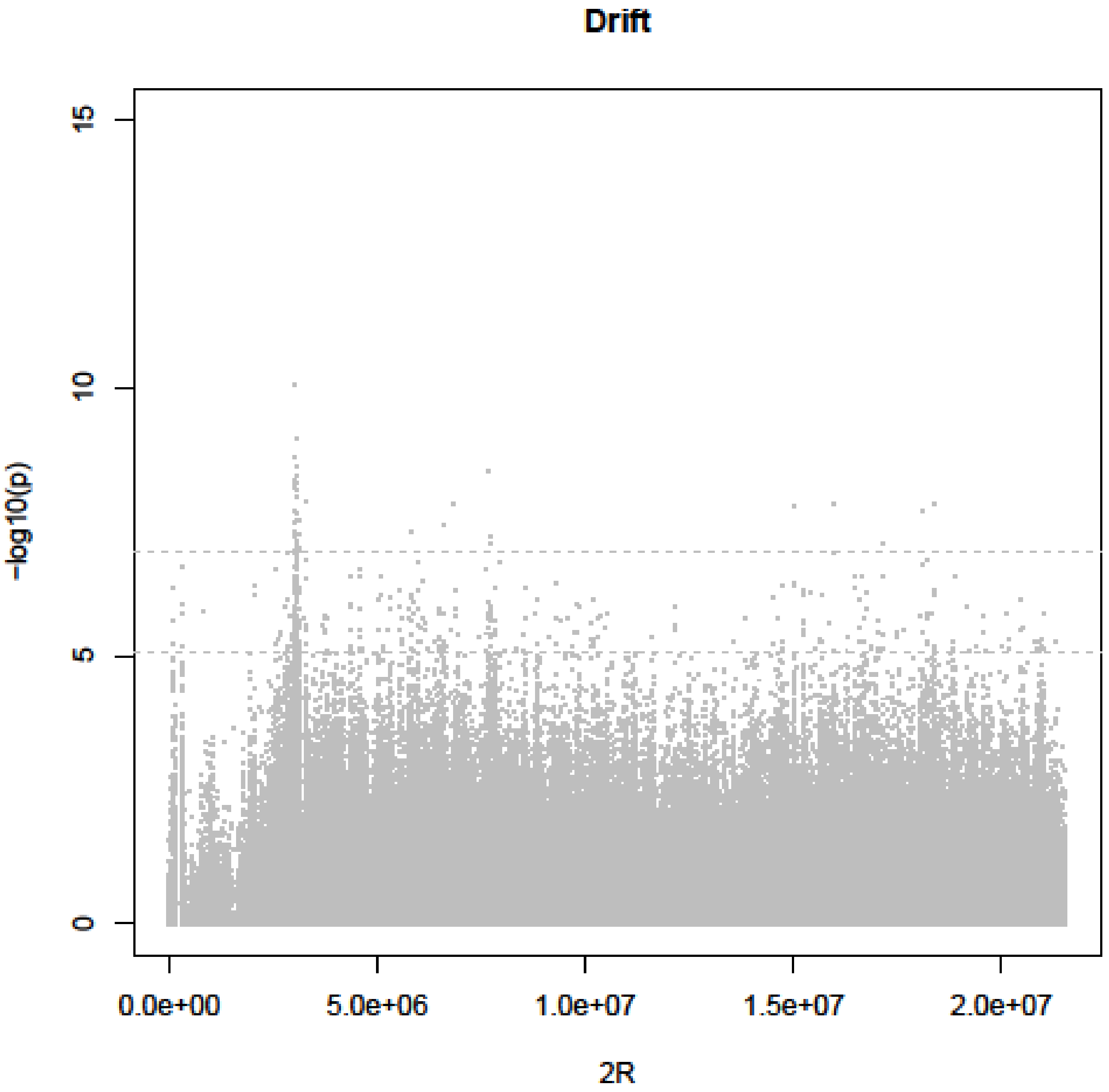

Supplemental figure 2c. We quantified genetic drift by performing CMH tests between replicate 1 and 2 from each of the three thermal regimes. The resulting distribution of p-values across the genome is a good representation of the pattern that we may expect due to drift and other sources of structure in our data. Using this distribution we determined two cut-offs used to detect segments of the genome where frequency changes between two selection regimes are consistent enough that we can interpret it as a product of selection. The 0.001% and 0.0001% percentile corresponded to -log10(p) thresholds of 5.06 and 6.95, respectively. Each chromosomal segment is shown separately (here chromosome 2R). X-axis represent position on chromosome.

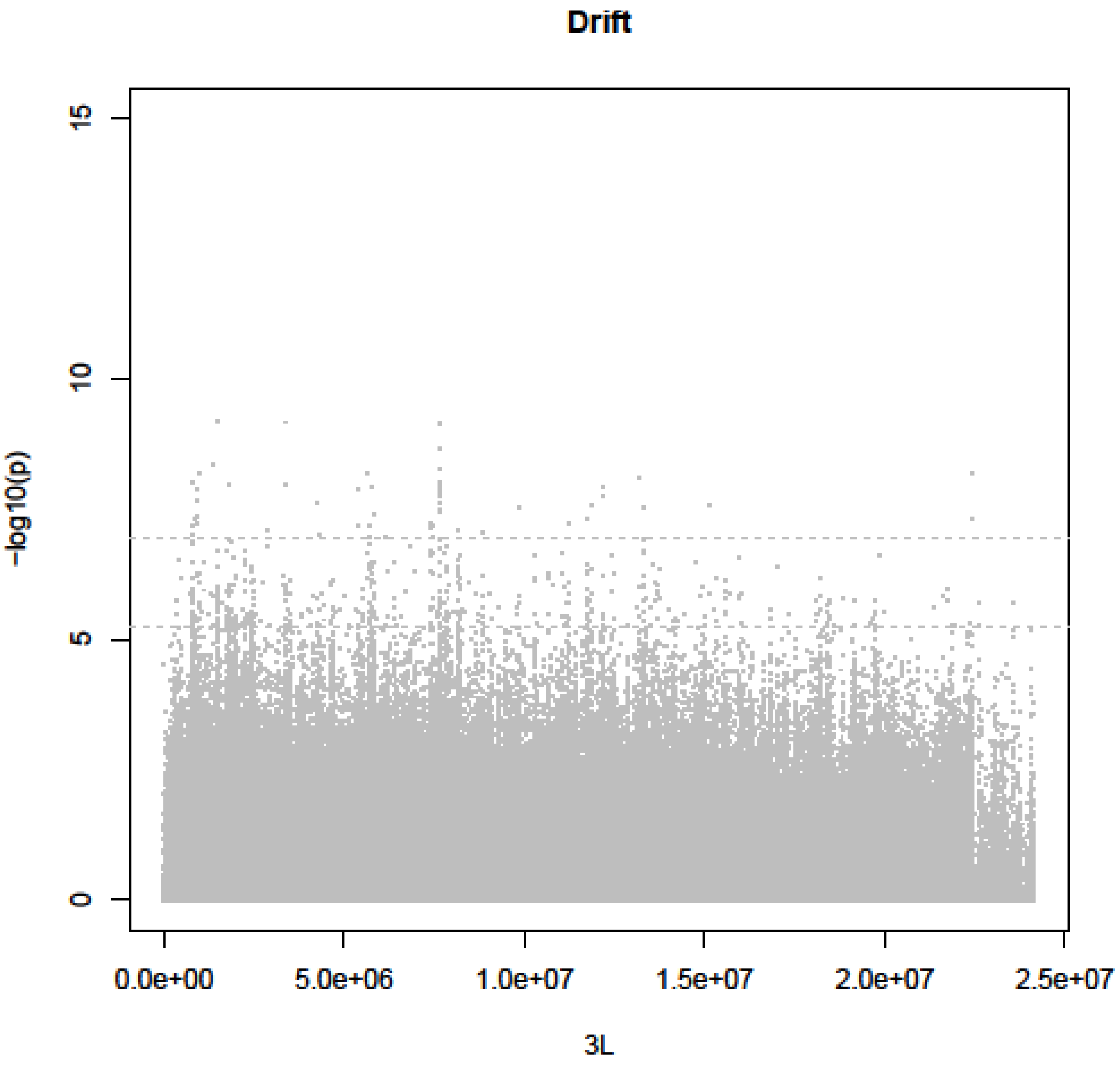

Supplemental figure 2d. We quantified genetic drift by performing CMH tests between replicate 1 and 2 from each of the three thermal regimes. The resulting distribution of p-values across the genome is a good representation of the pattern that we may expect due to drift and other sources of structure in our data. Using this distribution we determined two cut-offs used to detect segments of the genome where frequency changes between two selection regimes are consistent enough that we can interpret it as a product of selection. The 0.001% and 0.0001% percentile corresponded to -log10(p) thresholds of 5.23 and 6.96, respectively. Each chromosomal segment is shown separately (here chromosome 3L). X-axis represent position on chromosome.

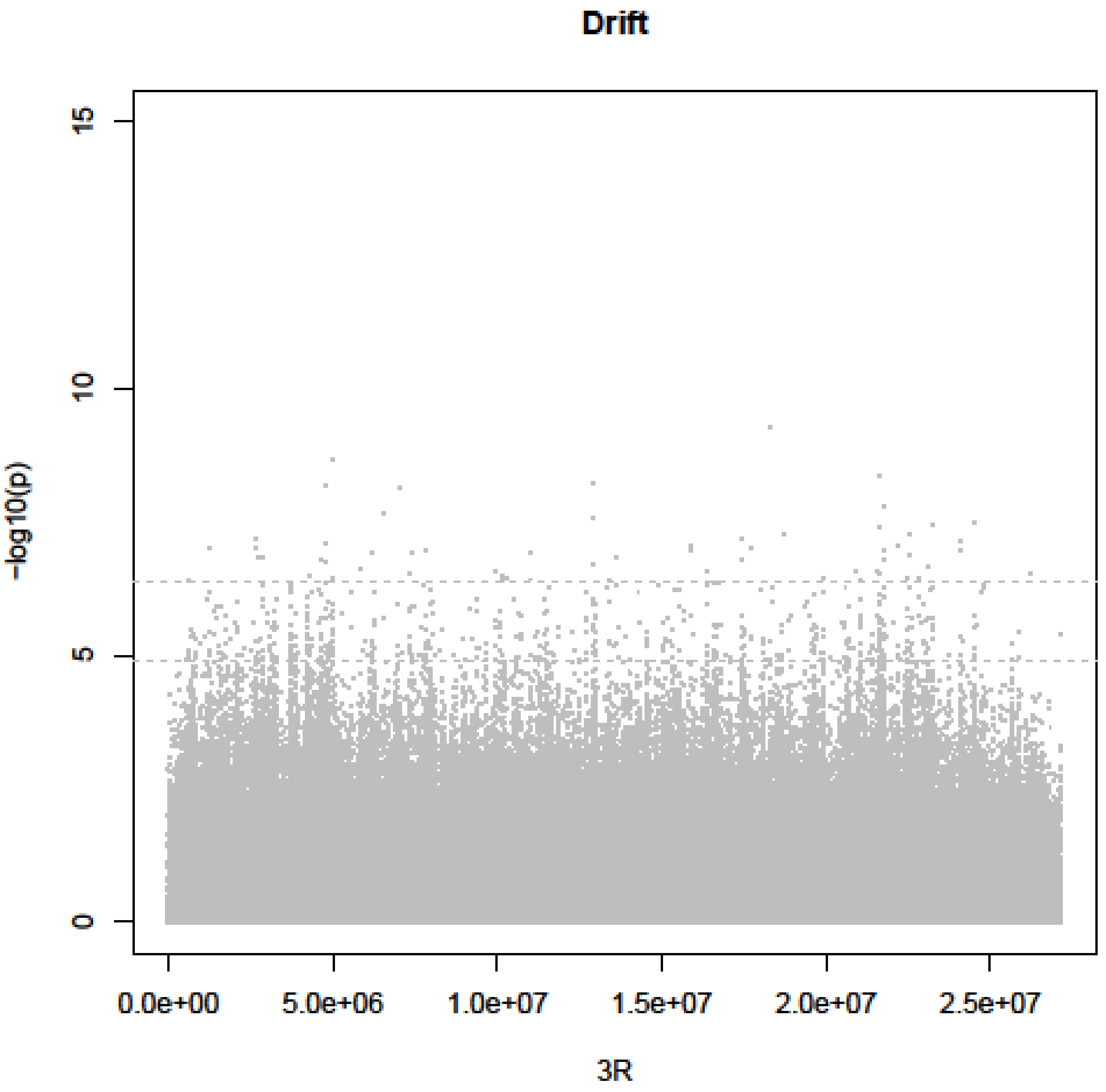

Supplemental figure 2e. We quantified genetic drift by performing CMH tests between replicate 1 and 2 from each of the three thermal regimes. The resulting distribution of p-values across the genome is a good representation of the pattern that we may expect due to drift and other sources of structure in our data. Using this distribution we determined two cut-offs used to detect segments of the genome where frequency changes between two selection regimes are consistent enough that we can interpret it as a product of selection. The 0.001% and 0.0001% percentile corresponded to -log10(p) thresholds of 4.89 and 6.36, respectively. Each chromosomal segment is shown separately (here chromosome 3R). X-axis represent position on chromosome.

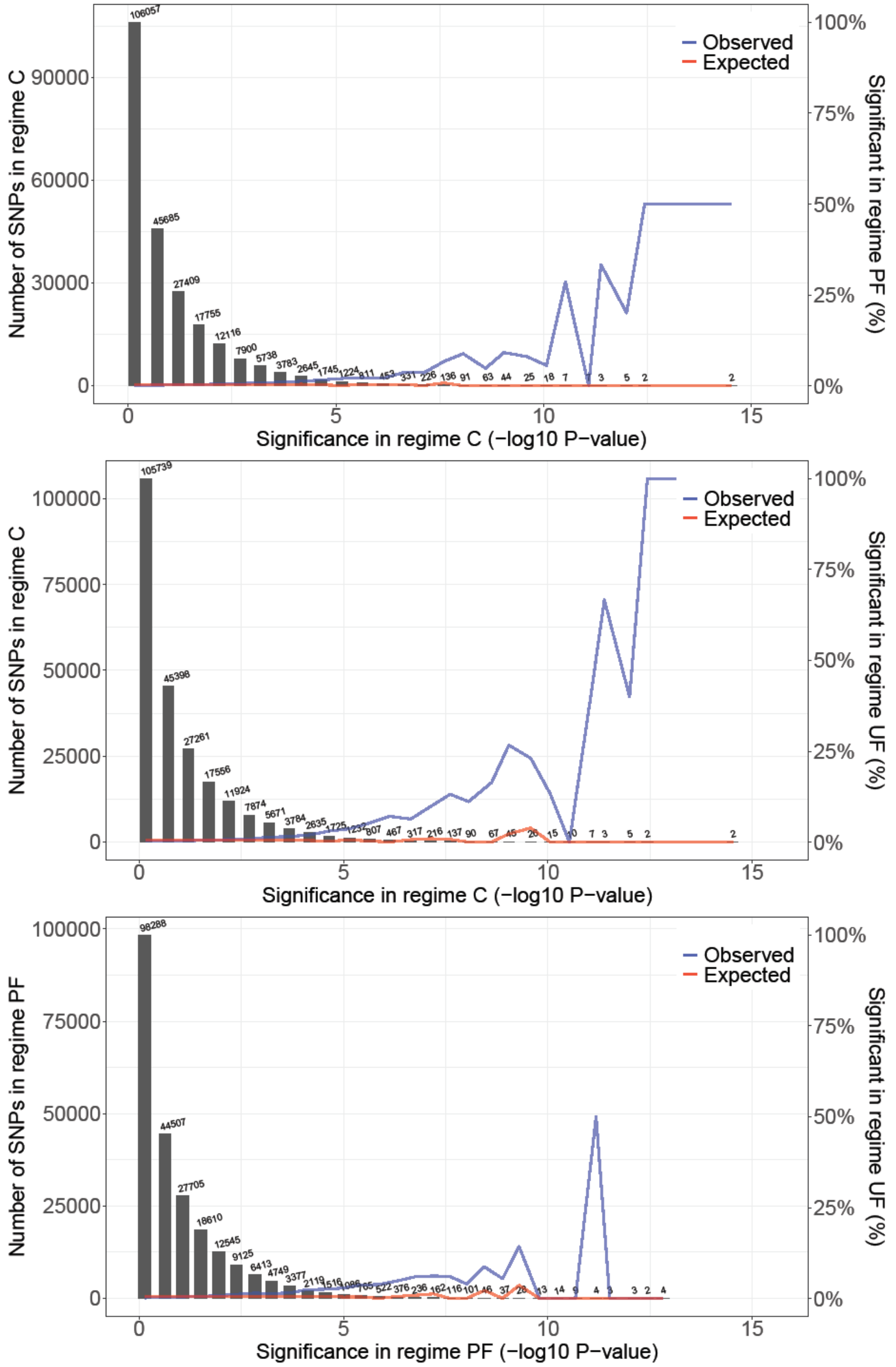

Supplemental figure 3a. Pairwise overlap of significant SNPs among thermal regimes (here chromosome X). Plot shows the distribution of SNP significance (–log10(p-values)) in one regime (grey bars), the observed proportion of SNPs that overlap with significant SNPs in the second regime, and the proportion of SNPs with randomized significance in the second regime that overlap (null-expectation). There is a consistent signal of overlap of SNPs with low p-values (in a small number of SNPs) in all pairwise comparisons. Thus, of the few SNPs with low p-values (high –log10(p)) in one selection regime a larger proportion than expected by chance also have low p-values in the other selection regimes.

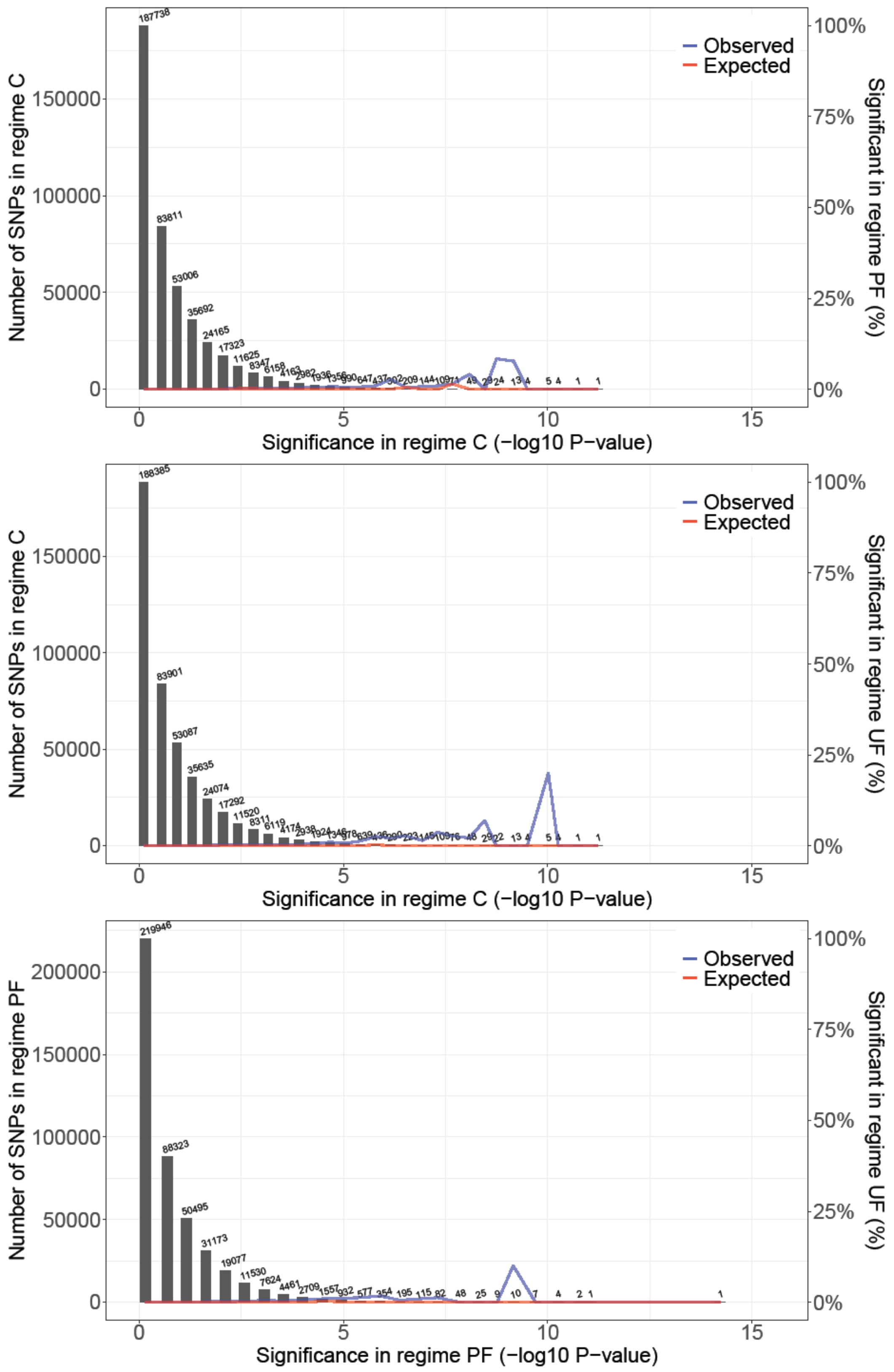

Supplemental figure 3b. Pairwise overlap of significant SNPs among thermal regimes (here chromosome 2L). Plot shows the distribution of SNP significance (–log10(p-values)) in one regime (grey bars), the observed proportion of SNPs that overlap with significant SNPs in the second regime, and the proportion of SNPs with randomized significance in the second regime that overlap (null-expectation). There is a consistent signal of overlap of SNPs with low p-values (in a small number of SNPs) in all pairwise comparisons. Thus, of the few SNPs with low p-values (high –log10(p)) in one selection regime a larger proportion than expected by chance also have low p-values in the other selection regimes.

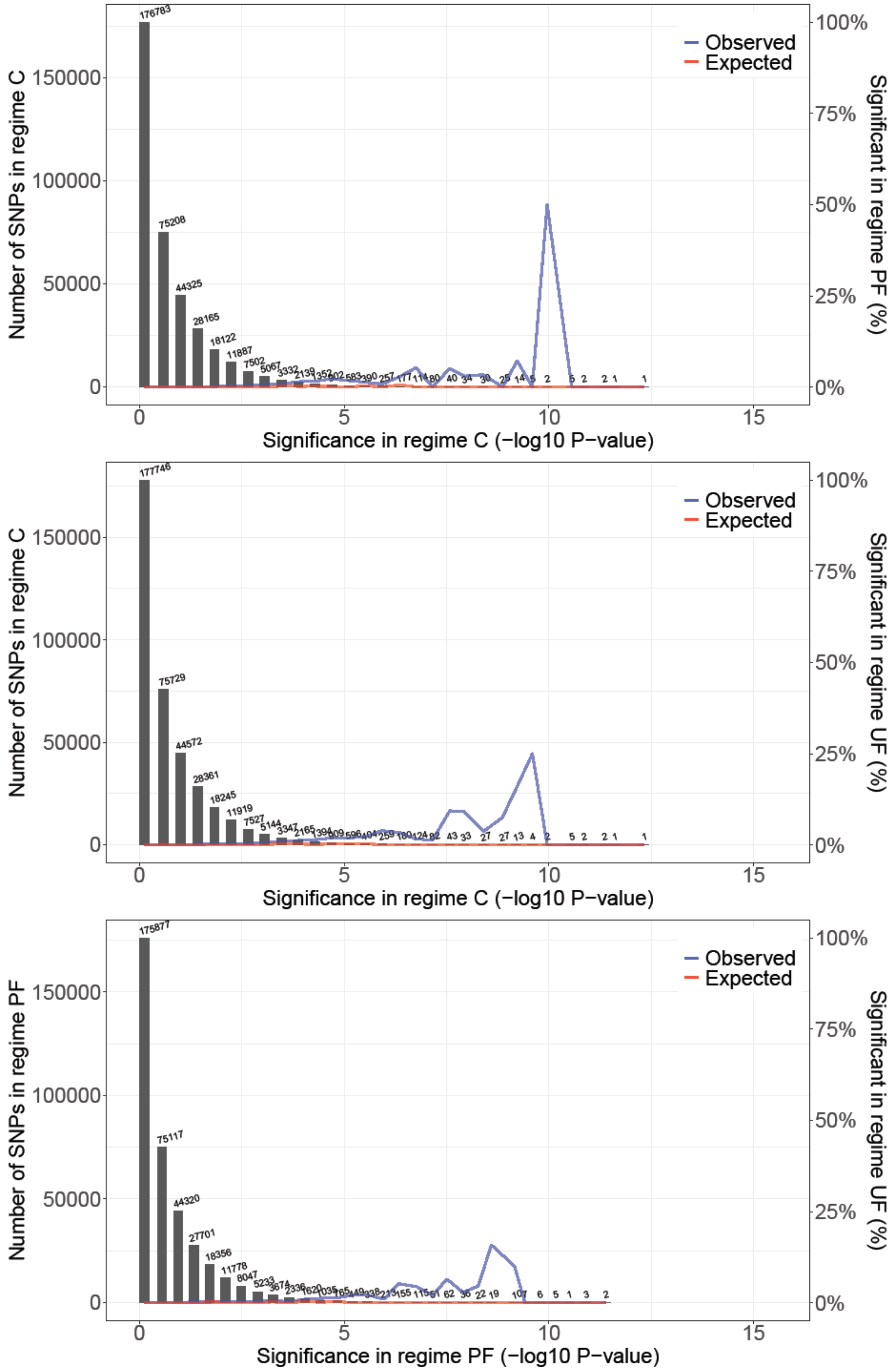

Supplemental figure 3c. Pairwise overlap of significant SNPs among thermal regimes (here chromosome 2R). Plot shows the distribution of SNP significance (–log10(p-values)) in one regime (grey bars), the observed proportion of SNPs that overlap with significant SNPs in the second regime, and the proportion of SNPs with randomized significance in the second regime that overlap (null-expectation). There is a consistent signal of overlap of SNPs with low p-values (in a small number of SNPs) in all pairwise comparisons. Thus, of the few SNPs with low p-values (high –log10(p)) in one selection regime a larger proportion than expected by chance also have low p-values in the other selection regimes.

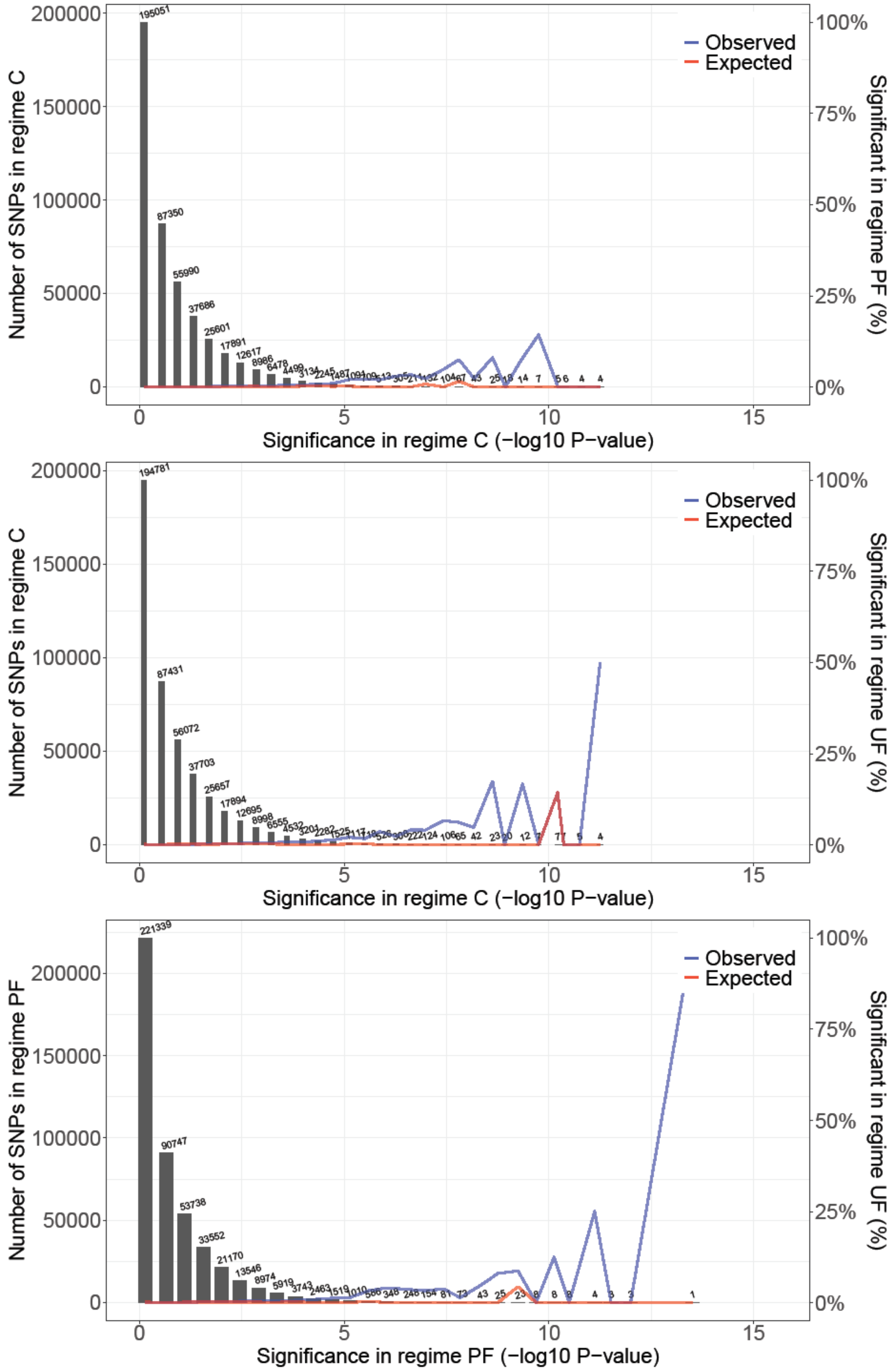

Supplemental figure 3d. Pairwise overlap of significant SNPs among thermal regimes (here chromosome 3L). Plot shows the distribution of SNP significance (–log10(p-values)) in one regime (grey bars), the observed proportion of SNPs that overlap with significant SNPs in the second regime, and the proportion of SNPs with randomized significance in the second regime that overlap (null-expectation). There is a consistent signal of overlap of SNPs with low p-values (in a small number of SNPs) in all pairwise comparisons. Thus, of the few SNPs with low p-values (high –log10(p)) in one selection regime a larger proportion than expected by chance also have low p-values in the other selection regimes.

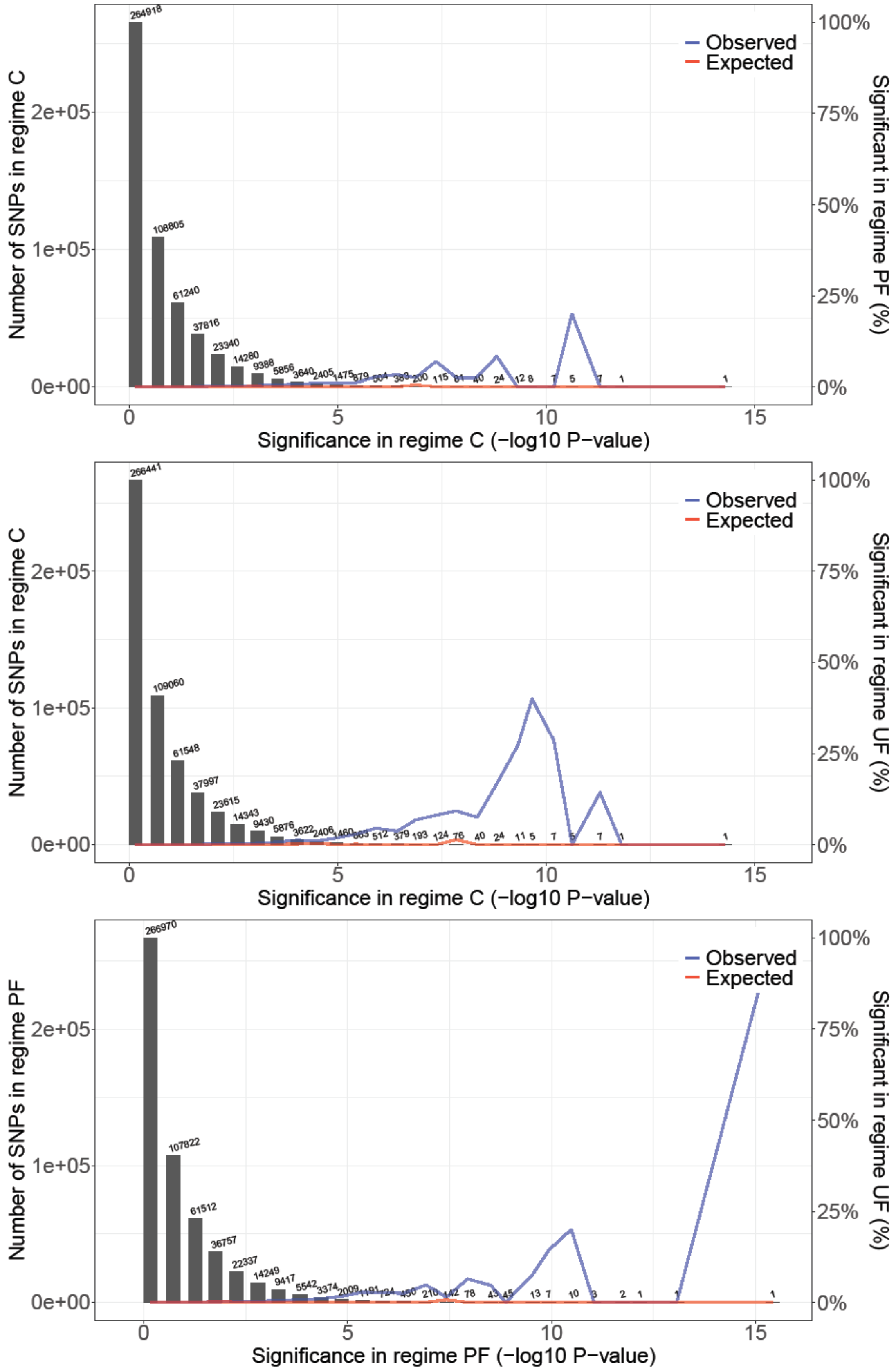

Supplemental figure 3e. Pairwise overlap of significant SNPs among thermal regimes (here chromosome 3R). Plot shows the distribution of SNP significance (–log10(p-values)) in one regime (grey bars), the observed proportion of SNPs that overlap with significant SNPs in the second regime, and the proportion of SNPs with randomized significance in the second regime that overlap (null-expectation). There is a consistent signal of overlap of SNPs with low p-values (in a small number of SNPs) in all pairwise comparisons. Thus, of the few SNPs with low p-values (high –

log10(p)) in one selection regime a larger proportion than expected by chance also have low p-values in the other selection regimes.

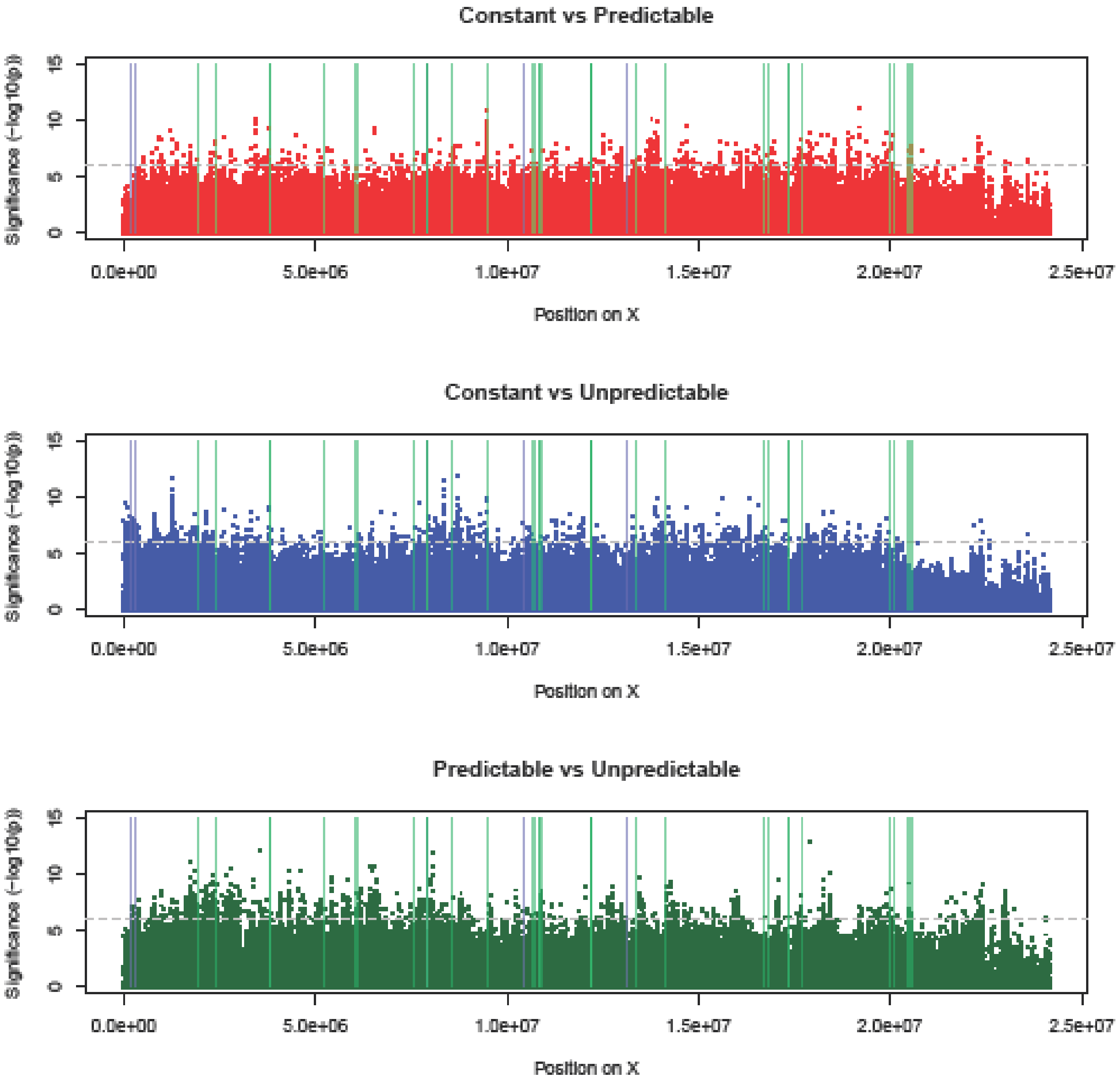

Supplemental figure 4a. Manhattan plots comparing pairs of selection regimes for each chromosomal segment (here chromosome X). Horizontal lines indicate two thresholds used to infer patterns of differentiation due to adaptation across selection regimes; 0.001% and 0.0001% percentile. SNPs with p-values above these thresholds can be considered evidence of divergence between selection regimes due to adaptation. Vertical lines indicate positions of loci with diverging gene expression levels (Manenti et al., 2018) (blue) or diverging protein expression levels (green).

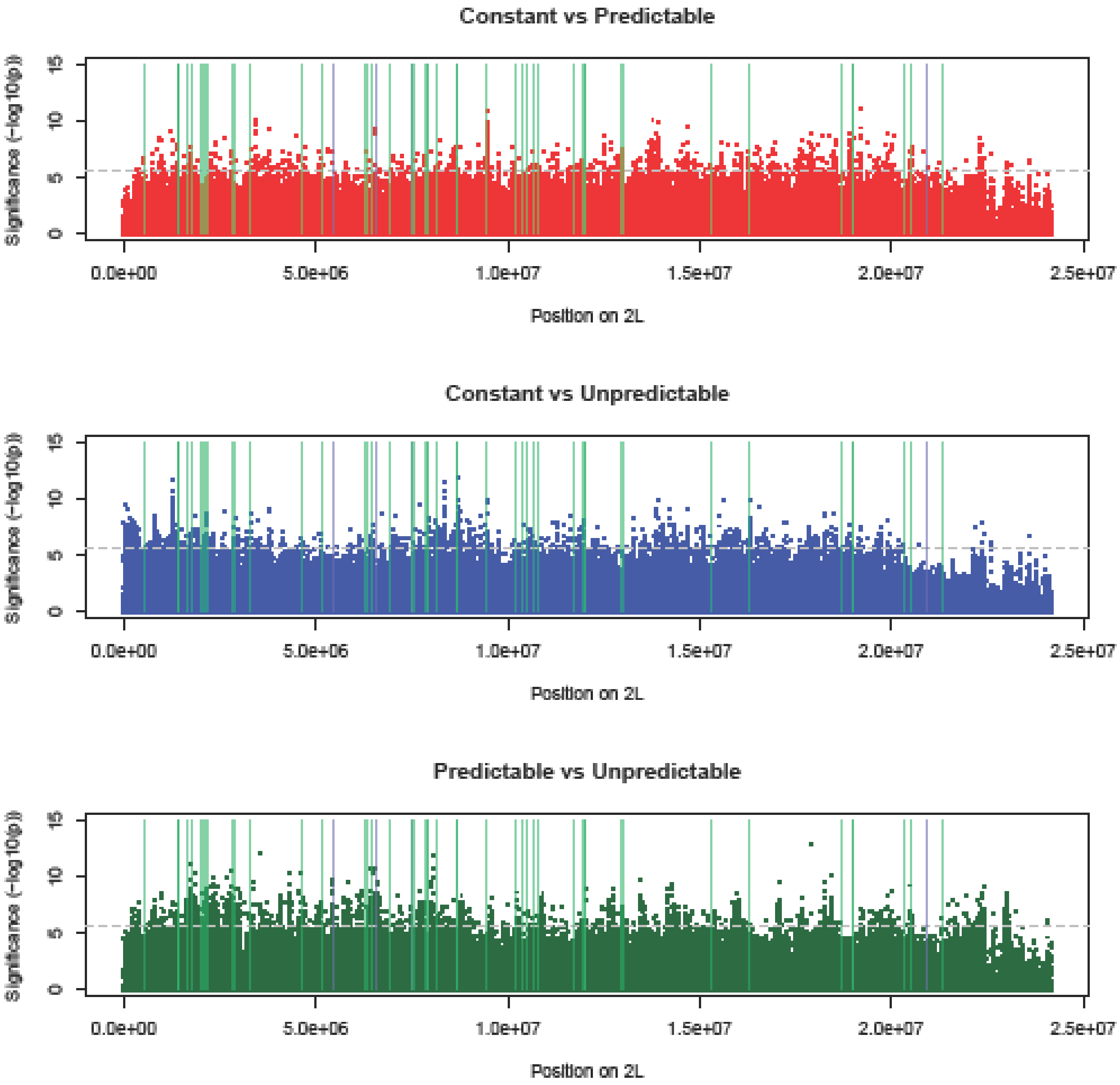

Supplemental figure 4b. Manhattan plots comparing pairs of selection regimes for each chromosomal segment (here chromosome 2L). Horizontal lines indicate two thresholds used to infer patterns of differentiation due to adaptation across selection regimes; 0.001% and 0.0001% percentile. SNPs with p-values above these thresholds can be considered evidence of divergence between selection regimes due to adaptation. Vertical lines indicate positions of loci with diverging gene expression levels (Manenti et al., 2018) (blue) or diverging protein expression levels (green).

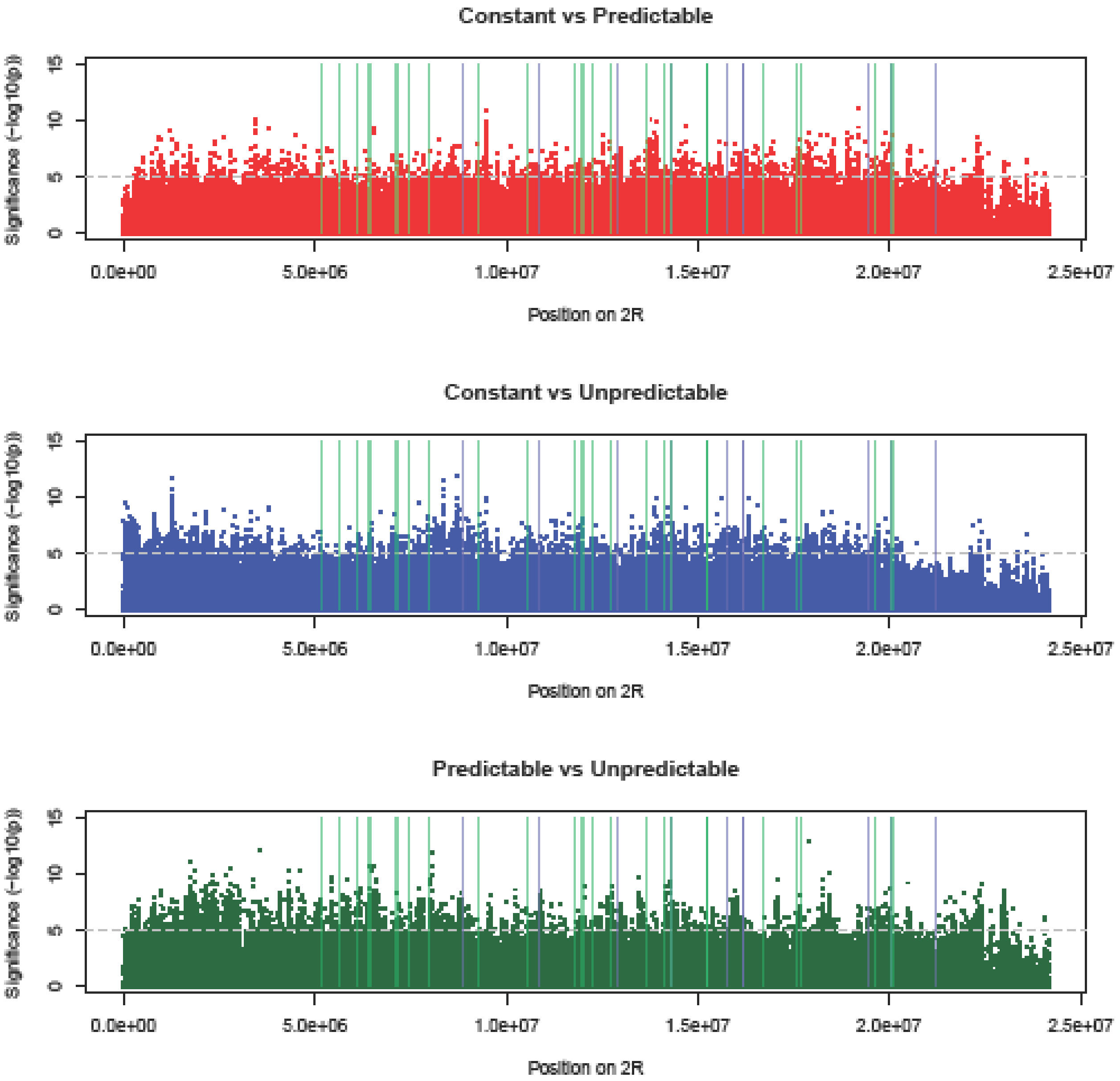

Supplemental figure 4c. Manhattan plots comparing pairs of selection regimes for each chromosomal segment (here chromosome 2R). Horizontal lines indicate two thresholds used to infer patterns of differentiation due to adaptation across selection regimes; 0.001% and 0.0001% percentile. SNPs with p-values above these thresholds can be considered evidence of divergence between selection regimes due to adaptation. Vertical lines indicate positions of loci with diverging gene expression levels (Manenti et al., 2018) (blue) or diverging protein expression levels (green).

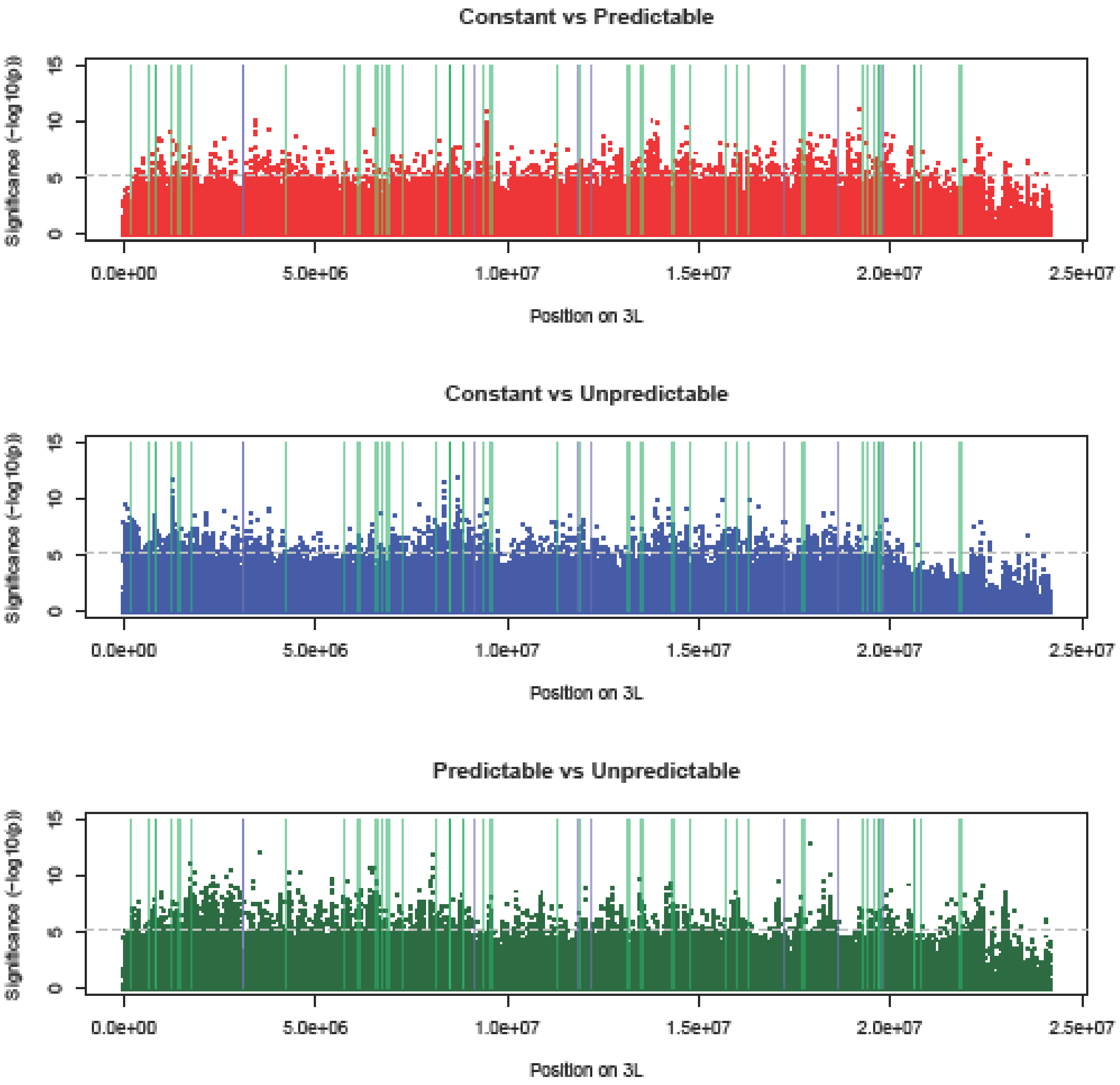

Supplemental figure 4d. Manhattan plots comparing pairs of selection regimes for each chromosomal segment (here chromosome 3L). Horizontal lines indicate two thresholds used to infer patterns of differentiation due to adaptation across selection regimes; 0.001% and 0.0001% percentile. SNPs with p-values above these thresholds can be considered evidence of divergence between selection regimes due to adaptation. Vertical lines indicate positions of loci with diverging gene expression levels (Manenti et al., 2018) (blue) or diverging protein expression levels (green).

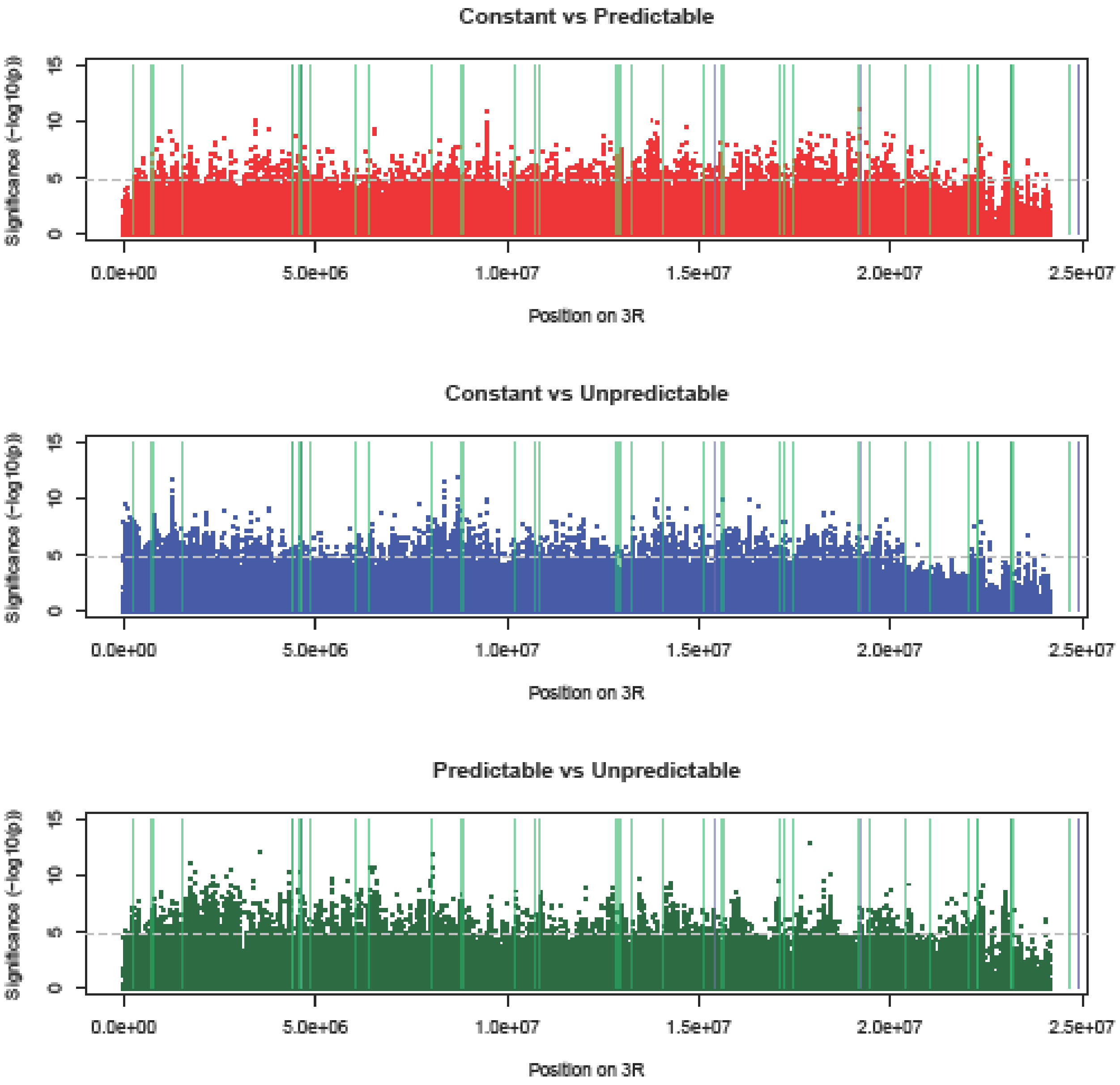

Supplemental figure 4e. Manhattan plots comparing pairs of selection regimes for each chromosomal segment (here chromosome 3R). Horizontal lines indicate two thresholds used to infer patterns of differentiation due to adaptation across selection regimes; 0.001% and 0.0001% percentile. SNPs with p-values above these thresholds can be considered evidence of divergence between selection regimes due to adaptation. Vertical lines indicate positions of loci with diverging gene expression levels (Manenti et al., 2018) (blue) or diverging protein expression levels (green).

References

Manenti, T., Loeschcke, V., Sørensen, J.G., 2018. Constitutive up-regulation of Turandot genes rather than changes in acclimation ability is associated with the evolutionary adaptation to temperature fluctuations in *Drosophila simulans*. Journal of Insect Physiology 104, 40-47.
